# Altered energy metabolism in Fatal Familial Insomnia cerebral organoids is associated with astrogliosis and neuronal dysfunction

**DOI:** 10.1371/journal.pgen.1010565

**Published:** 2023-01-19

**Authors:** Simote T. Foliaki, Anna Smith, Benjamin Schwarz, Eric Bohrnsen, Catharine M. Bosio, Katie Williams, Christina D. Orrú, Hailey Lachenauer, Bradley R. Groveman, Cathryn L. Haigh

**Affiliations:** 1 Laboratory of Persistent Viral Diseases, National Institute of Allergy and Infectious Diseases, Division of Intramural Research, Rocky Mountain Laboratories, National Institutes of Health, Hamilton, Montana, United States of America; 2 Laboratory of Bacteriology, National Institute of Allergy and Infectious Diseases, Division of Intramural Research, Rocky Mountain Laboratories, National Institutes of Health, Hamilton, Montana, United States of America; 3 Research Technologies Branch, National Institute of Allergy and Infectious Diseases, Division of Intramural Research, Rocky Mountain Laboratories, National Institutes of Health, Hamilton, Montana, United States of America; Case Western Reserve University School of Medicine, UNITED STATES

## Abstract

Fatal familial insomnia (FFI) is a rare neurodegenerative disease caused by a dominantly inherited single amino acid substitution (D178N) within the prion protein (PrP). No in vitro human brain tissue model for this disease has previously been available. Consequently, how this mutation exerts its damaging effect on brain cells is still unknown. Using CRISPR-Cas9 engineered induced pluripotent stem cells, we made D178N cerebral organoids and compared these with isotype control organoids. We found that, in the absence of other hallmarks of FFI, the D178N organoids exhibited astrogliosis with cellular oxidative stress. Abnormal post-translational processing of PrP was evident but no tissue deposition or propagation of mis-folded PrP isoforms were observed. Neuronal electrophysiological function was compromised and levels of neurotransmitters, particularly acetylcholine and GABA, altered. Underlying these dysfunctions were changes in cellular energy homeostasis, with substantially increased glycolytic and Krebs cycle intermediates, and greater mitochondrial activity. This increased energy demand in D178N organoids was associated with increased mitophagy and depletion of lipid droplets, in turn resulting in shifts of cellular lipid composition. Using a double mutation (178NN) we could confirm that most changes were caused by the presence of the mutation rather than interaction with PrP molecules lacking the mutation. Our data strongly suggests that shifting biosynthetic intermediates and oxidative stress, caused by an imbalance of energy supply and demand, results in astrogliosis with compromised neuronal activity in FFI organoids. They further support that many of the disease associated changes are due to a corruption of PrP function and do not require propagation of PrP mis-folding.

## Author summary

Fatal familial insomnia is a devastating disease characterized by progressively worsening sleep until the brain and body deterioration result in death. Degeneration of brain cells is associated with accumulation of mis-folded proteins called prions. The mis-folding of the prions is caused by a single point mutation, D178N, in the gene encoding the normal precursor protein of the prions, the prion protein. Herein, we have used a human brain model to investigate the how this mutation causes brain cell damage. Our findings show that the mutation causes brain cells to dysfunction without the need for mis-folding into prions. Brain cell dysfunction was associated with stress and abnormal metabolism in such a way as to change critical compounds needed for the brain cells to function correctly. The changes correlate with enhanced wakefulness, which could cause the sleep disturbance. Our data support that many of the disease associated changes are caused by the fatal familial insomnia D178N mutation corrupting prion protein function.

## Introduction

Prion diseases encompass a family of rare and incurable neurodegenerative diseases. About 15% of prion disease cases are genetic; caused by autosomal dominant mutations within the *PRNP* gene encoding the prion protein (PrP). These diseases include genetic Creutzfeldt Jakob Disease (CJD), Gerstmann-Sträussler Scheinker disease (GSS), and Fatal Familial Insomnia (FFI). FFI is caused by a point mutation that substitutes aspartic acid with asparagine at codon 178 (D178N) of the *PRNP* gene in cis with a methionine at the codon 129 polymorphism site [1]. Interestingly, if the codon 129 polymorphism is a valine, the D178N mutation instead causes genetic CJD. The FFI mutation has a high penetrance, thus the carriers are likely to develop the disease [2]. The age of onset is generally between 45–50, and the disease phenotypes include deteriorating insomnia and abnormal muscle function leading to death [1, 3].

As is true of the whole family of prion diseases, onset of FFI correlates with detection of misfolded PrP species or prions, which are specific biomarkers of prion disease. Prions can be detected in some mouse models of FFI, but only at the clinical disease stage [4–6], and what causes the switch from normally folded PrP to mis-folded prions is unknown. Prions are insoluble, protease-resistant (PrP^Res^), and transmissible [4–6]. The transmissible nature of prions means that they can self-propagate or seed the conversion of normal PrP into prions. This seeding ability allows the disease to spread through the brain and can be measured by highly sensitive and specific assays such as Real-time Quaking-Induced Conversion (RT-QuIC) and Protein Misfolding Cyclic Amplification (PMCA) [7–9]. The D178N mutation does not result in steady-state production of prions in cell culture models; however, it directly alters the biochemical properties of PrP, producing heavily glycosylated PrP [4–6]. This change was also evident in PrP^Res^ from a terminal FFI human brain [10].

FFI alters numerous molecular pathways that are essential for survival, complicating the mechanistic insights into how FFI specifically damages the brain. Terminal FFI brains have been shown to have altered regulation of transcription, RNA splicing, protein synthesis and folding, protein transport, mitochondrial function, and oxidation-reduction [11]. Disrupted mitochondrial subunits and increased oxidative stress defense have been found in astrocytes in terminal FFI mediodorsal thalamus relative to age-matched healthy controls [12]. The evolution of these dysfunctions during the disease progression, whether they start before the disease onset, is unknown.

Cerebral organoids (COs) have been used to model various genetic neurological disorders, including Alzheimer’s Disease, Parkinson’s Disease, Trisomy 21, and genetic CJD [13–17]. Our group has also demonstrated successful infection of COs with prions from terminal stage sporadic CJD [15]. When we examined the *PRNP* E200K CJD-causing mutation, we found COs differentiated from carriers with this mutation do not produce pathogenic prions [16], however as the organoids age, a dysregulated phenotype emerges. In healthy organoids, after approximately five months of age cell populations have matured sufficiently that they exhibit complex neural oscillation and network communication, contain fewer stem cells, and have populated more astrocytes [18]. For the E200K genetic CJD COs, reduced neuronal function and neural network oscillation are associated with changed synapse composition and neurotransmitter levels, indicating the mutation causes neuronal dysfunction [17].

To study the impact of the FFI D178N mutation in human brain tissue, we compared human COs with the D178N mutation in the *PRNP* gene with isogenic control COs. We found that the mutation does not cause spontaneous prion production. However, an astrogliosis was present, which appeared associated with increased oxidative stress and substantial shifts in energy pathway utilization within the COs. Increased mitophagy was evident and greater lipid catabolism from lipid droplets correlated with global shifts in organoid lipid composition. Further, our data indicate that an imbalance of energy supply and demand results in impaired neuronal activity.

## Results

### Generation of D178N CO cultures

**Fig 1:**
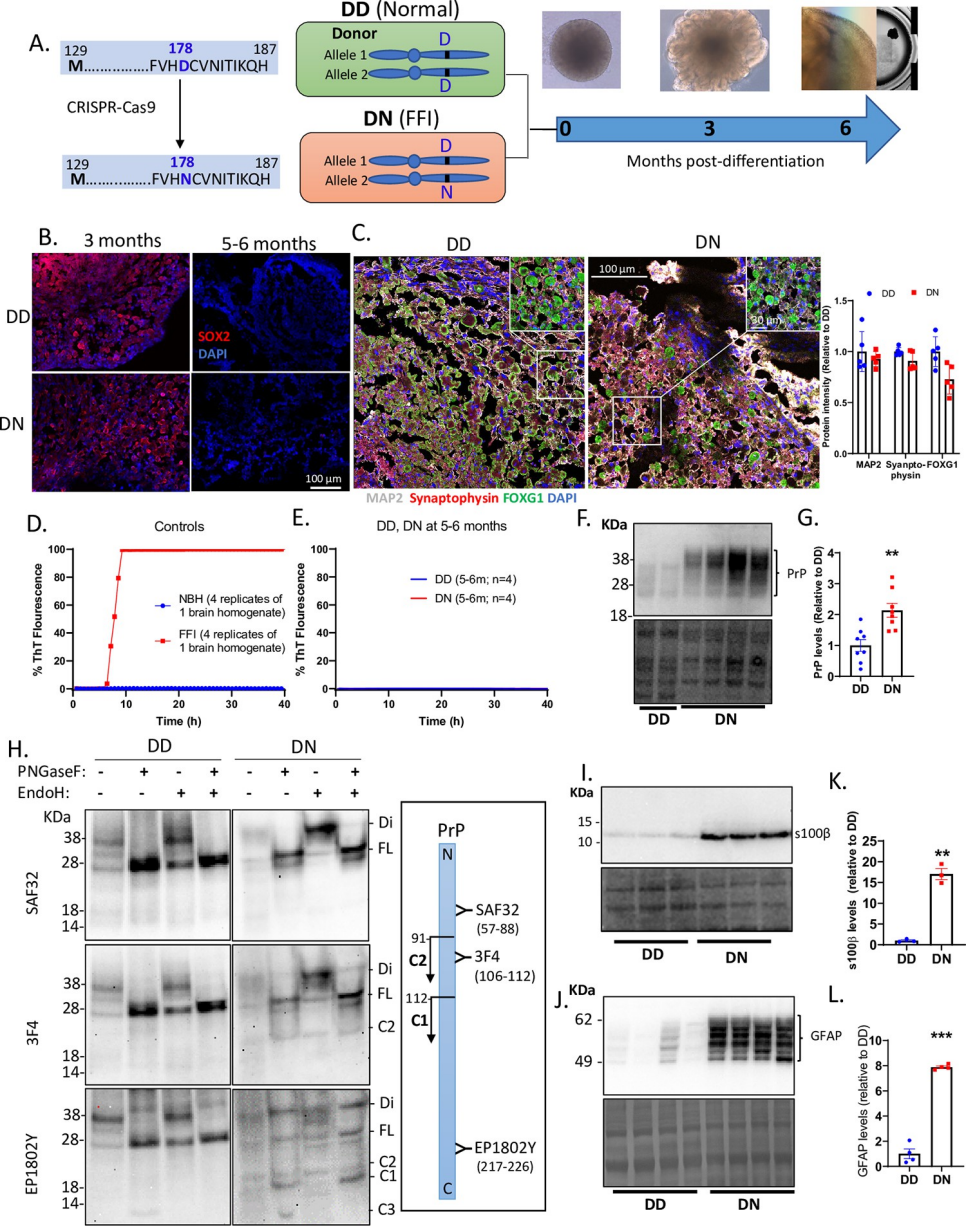
Generation of cerebral organoids and prion disease pathology. (A) A schematic diagram of how the D178N mutation was introduced into the PRNP gene of iPSCs donated by a healthy donor with a 178D and 129M in both alleles of PRNP gene (DD) to produce a heterozygous mutant line (DN). The cerebral organoids were generated from the DD and DN iPSCs and we studied them when they matured at ~5–6 months old. (B) Immunofluorescence images of the organoids at ~3 and ~6 months old labeled with SOX2. (C) Immunofluorescence images of the organoids at ~5 months old labeled with neuronal markers synaptophysin and MAP2, and cortical marker FOXG1. Insets show magnified images. The right panel is the quantification of those markers. (D, E) RT-QuIC assessment of prion seeding activity in control homogenates from a terminally ill FFI brain and a healthy brain (D), and in lysates from DD and DN COs at ~5–6 months old (E). (F) Western blot showing PrP in lysates of DD and DN COs using 3F4 prion antibody (upper panel) and the Coomassie stain for total protein (lower panel). (G) Quantification of the PrP levels after normalizing to the total protein. (H) Western blot detection of PrP using SAF32, 3F4, and EP1802Y antibodies in DD and DN lysates before (-) and after treatment (+) with either/both PNGaseF and Endoglycosidase H. Acronyms listed on the right indicate PrP species/fragments, including those that are di-glycosylated (Di), Full length (FL), and cleavage C1/C2/C3 fragments. The right panel is a schematic diagram of PrP (N to C terminus) with the binding sites of the PrP antibodies used and the cleavage sites that yield the C1 and C2 fragments. (I) Western blotting for s100β (top left panel) and the Coomassie stain for total protein (bottom left panel). (J) Western blot detection of GFAP (top left panel) and the Coomassie stain for the total protein (bottom left panel). (K, L) Quantification of the s100β and GFAP levels after normalizing to the total protein. (F, H, I, J). For each blot molecular weight markers are on the left. (C, G, K, L) Each dot is an “n” representing an organoid. Data are presented as mean ±SEM. (G, K, L) Statistically compared by Welch’s t-test. ** p<0.01, ***p<0.001.

The *PRNP* D178N mutation was introduced into a no-known-disease iPSC line by CRISPR-Cas9 gene editing, in which the aspartic acid (D) at codon 178 was substituted with an asparagine (N) (S1A Fig). This produced an iPSC line that was heterozygous (DN) for the mutation and an isogenic control line (DD). CO cultures were differentiated from these cells and grown to 5–6 months old–a time point corresponding with developed neurophysiological function ([17]; See the schematic diagram in Fig 1A). To ensure that all COs underwent normal maturation processes, we assessed cellular levels of SOX2, a marker of stem cells at 3 versus 5–6 months old. In both CO lines, the SOX2 was reduced at 5–6 months relative to 3 months (Fig 1B), demonstrating depleted stem cells at 5–6 months. At 4–5 months, both lines expressed neuronal markers synaptophysin and MAP2, which co-expressed with FOXG1 (Fig 1C), a transcription factor essential for cerebral cortex patterning and layering [19]. These showed that the COs were heavily populated with neurons resembling the human cerebral cortex. Overall, COs with the D178N mutation underwent normal maturation processes with no observed differences from the isogenic control COs. Hence, the rest of this study will be focused on the mature organoids (5–6 months old).

### D178N COs do not demonstrate spontaneous prion propagation but do display astrogliosis

To determine if the DN COs would develop a spontaneous prion infection, we looked for the classical biochemical hallmarks of replicating prions; insoluble and proteinase-K (PK) resistant PrP and prion seeding activity. No prion seeding activity was detectable in the DN COs by the highly sensitive RT-QuIC assay (Fig 1D and 1E). This seeding assay also confirmed that the DN COs were not accumulating other proteins associated with neurodegeneration including mis-folded Tau [20] or alpha-synuclein [21]. Additionally, increased amyloid-beta was not observed by western blotting (S2C and S2D Fig), however, the amyloid precursor protein appeared to have increased cleavage in the DN relative to the DD COs (S2E Fig). Correspondingly, DN COs contained no PrP species that were sarkosyl insoluble or PK-resistant (S2 Fig). Interestingly, the protein levels of total PrP were significantly higher in the DN than the DD (Fig 1F and 1G). As reported previously in mouse models of FFI [4, 5], we observed increased levels of di-glycosylated PrP in the DN COs (Fig 1F), suggesting that the mutation altered the post-translational modification of PrP. We assessed this by digesting PrP with Endoglycosidase H and PNGase F enzymes to remove glycans added in the ER (Endoglycosidase H-sensitive or immature PrP) and total glycans, respectively. Both the DD and DN showed Endoglycosidase H resistant PrP species (Fig 1H), which were mostly de-glycosylated by PNGaseF into FL PrP (Fig 1H lanes 2, 4, 6, 8). We also observed increased PrP fragmentation in the DN COs compared with the DD COs, including C1 and C2 fragments (Fig 1H). These results showed that most PrP species in the DN were mature and not pathogenic; however, they were expressed at higher levels, with different glycosylation and fragmentation from normal PrP. DN COs did recapitulate one hallmark of prion disease, astrogliosis, showing significantly higher levels of S100Beta and GFAP (Fig 1I–1L). While we found no pathogenic prions in the DN COs, the increased protein level and fragmentation of PrP coupled with the astrogliosis suggests they are undergoing cellular stress.

### D178N alters neuronal population network communication

**Fig 2:**
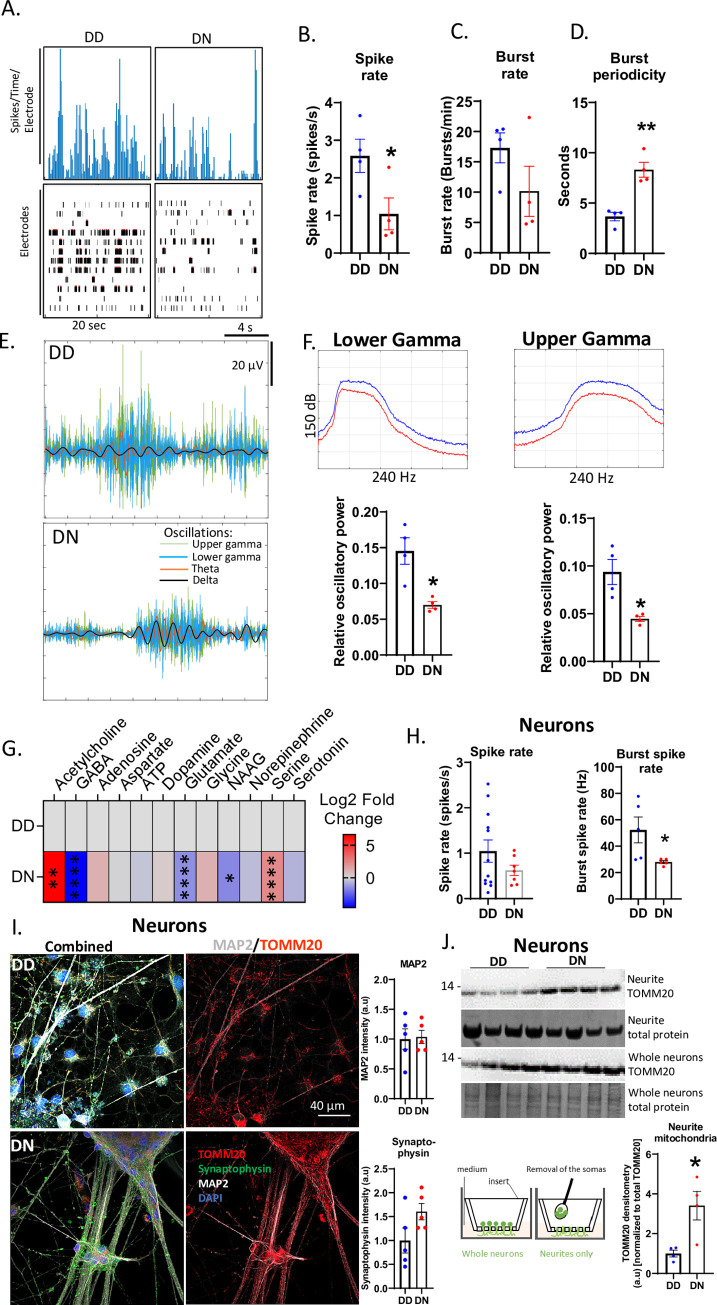
Effects of D178N mutation on the neuronal population network communication. (A) Representative neuronal firing frequency and raster plots recorded on ~6-month-old DD and DN organoids. (B-D) Comparison of the Spike (firing) rate (B), burst rate (C), and burst periodicity (D) in the DN to the DD organoids by Welch’s t test. (E) Representative delta, theta, and lower and upper gamma oscillations embedded in the neuronal population electrical signaling of DD and DN organoids. (F) Relative oscillatory powers of lower and upper gamma oscillation estimated by Welch’s power spectral density (top panels representatives plots) and compared between CO lines by Welch’s t test (bottom panels). (G) Log_2_ Fold changes in the levels of DN neurotransmitters relative to the DD COs with the degrees of statistical significance determined by a Multiple Student’s t test (n = 5 organoids per group). Neurotransmitter levels were measured by LCMS. Raw data are in the **S1 Dataset** Metabolomics source data file. (H) The spike rate and burst spike rate of DD and DN monolayer neurons, statistically compared between cell lines by Welch’s t test. (I) Representative immunofluorescence (IF) images of DD (top panels) and DN (bottom panels) monolayer neurons labeled with TOMM20, synaptophysin, MAP2, and DAPI. The left panels show images overlaid with all the stains, middle panels show TOMM20 and MAP2, and right panels are quantifications of MAP2 and synaptophysin from IF images represented in left panels. (J) Western blotting analysis of the neurite TOMM20 levels relative to the total TOMM20 in whole neurons after normalizing to total protein stained by Coomassie. The mean TOMM20 was compared between DD and DN by Mann-Whitney test. The schematic image at the bottom left shows how neurites were separated from the somas (B-G) Each dot is an “n” representing an organoid. All data were obtained from 5–6-month-old COs. (H, I, J) Each “n” represents a chamber of neurons. Data are presented as mean ±SEM. * p<0.05, ** p<0.01.

FFI is associated with a progressive dysfunction of neuronal firing and network communication. To determine if the DN COs exhibited this phenotype, we assessed the synchronous neuronal population firing and bursting by multi-electrode arrays (MEA) and found that the DN COs exhibited fewer neuronal spikes (firings) and bursts, or longer burst periodicity than the control (Fig 2A–2D). This was confirmed by a significantly reduced relative oscillatory power of the gamma oscillations in the DN compared with the DD COs (Fig 2E and 2F). The oscillatory powers of slower oscillations like delta and theta were unaltered (Figs 2E and S3). The changes in neuronal network communication were linked to altered levels of some small-molecule neurotransmitters in the DN COs, including acetylcholine, GABA, glutamate, NAAG, and serine (Fig 2G), suggesting that the synthesis of these neurotransmitters was changed by the mutation. To determine if the reduced neuronal function was due to cell population shifts caused by the astrogliosis, forebrain neuronal cultures were differentiated from the DD and DN iPSCs. Similar results were obtained for these mono-cultures, although only the burst rate but not the spike rate was significantly reduced, suggesting the DN neurons have an intrinsic functional deficit (Fig 2H). The levels of MAP2 and synaptophysin were not different between the DD and DN neurons, demonstrating that the mutation did not reduce the dendritic spines and synapses (Fig 2I). However, analysis of the neurites of these neurons separated from the somas (Fig 2J schematic diagram) revealed significantly more mitochondria in the neurites of the DN neurons than the DD neurons (Fig 2I and 2J). Together, this supports that the altered neuronal network communication in DN COs is likely caused by changed synthesis, packaging, and release of small-molecule neurotransmitters at dendrites and synaptic terminals through processes that require mitochondria [20].

### D178N mutation enhances oxidative stress

**Fig 3:**
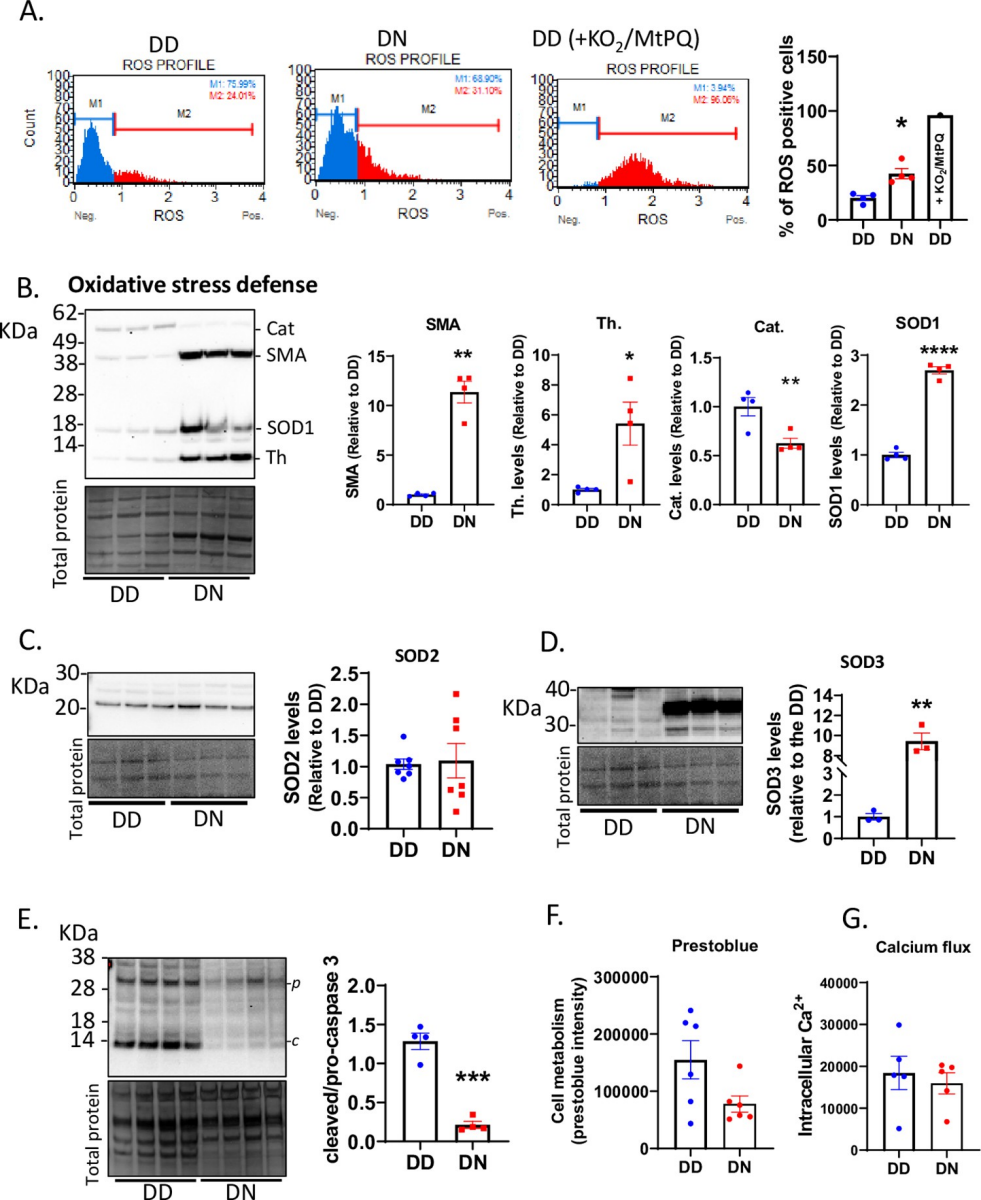
Oxidative stress is observed in the D178N COs. (A) Flow cytometry analysis of the number of cells that were ROS positive in the DD and DN COs and in a DD organoid treated with 1mM KO_2_ and 12.5μM Mito-Paraquat (MtPQ) to increase ROS (positive control). The left panels are representative plots and the right graph is the quantification expressed as the percentage of cells that are ROS positive. (B) Western blotting (Top left panel) for antioxidant markers, including catalase (Cat), SOD1, and thioredoxin (Th.), and for smooth muscle actin (SMA). The Coomassie stain loading control is the bottom left panel. The quantifications after normalizing to total protein are in the right panels. (C) Western blotting for SOD2 (top left panel), the Coomassie stain for total protein (bottom left panel), and the quantification of the levels of SOD2 after correcting to the total protein (right panel). (D) Western blotting for SOD3 (top left panel), the Coomassie stain for total protein (bottom left panel), and the quantification of the levels of SOD3 after correcting to the total protein (right panel). (E) Western blotting for pro-caspase 3 (*p*) and cleaved caspase 3 (*c*; top left panel), the Coomassie stain for the total protein (bottom left panel), and the quantification of the levels of cleaved/pro-caspase 3 after correcting to the total protein (right panel). (F) Prestoblue analysis of cell metabolism in the organoids at ~6 months old. (G) Levels of intracellular calcium in the organoids at ~6 months old. (B, C, D, E) Molecular weight markers are shown on the left of blots. (A-G) The average protein levels of DN were compared to the DD by Welch’s t tests. Each dot represents an organoid (“n”). Data were from 5-6-month-old COs and are presented as mean ±SEM. * p<0.05, ** p<0.01, *** p<0.001, ****p<0.0001.

Mitochondria are a major producer of reactive oxygen species (ROS) within cells and the astrogliosis indicated that the DN COs may be undergoing oxidative stress. To confirm this, we assessed cellular levels of ROS by flow cytometry and showed that the DN COs were producing significantly higher levels of ROS than the DD COs (Fig 3A). Assessment of anti-oxidant protein levels demonstrated that thioredoxin, SOD1, and SOD3 were significantly increased in the DN relative to the DD (Fig 3B and 3D), indicating that the DN cells were responding to cytosolic and extracellular oxidative stress. Smooth muscle actin, which was originally included as a loading control, also became significantly increased in the DN compared with the DD COs. This was likely due to increased expression in astrogliosis as shown previously [21]. SOD2 was unaltered, while catalase was reduced in the DN COs (Fig 3B and 3C), demonstrating less engagement of mitochondrial antioxidant activities. The increased activity of some of these antioxidants in the DN suggested that the cells were compensating for oxidative stress to remain viable. This was confirmed by the significant decrease in caspase 3 activity (cleaved caspase 3 levels) in the DN COs, possibly to reduce apoptotic cell death (Fig 3E). The unaltered levels of cell metabolism, detected using presto blue, and calcium flux in the DN relative to the DD COs demonstrated a relatively normal cell viability in the mutant COs (Fig 3F and 3G). Activation of the homeostatic survival mechanisms in the DN COs, especially by altering the levels of antioxidant enzymes in the cytosolic and extracellular compartments, appears sufficient to manage the oxidative stress and maintain their viability.

### D178N mutation alters metabolites essential for bioenergetics

**Fig 4:**
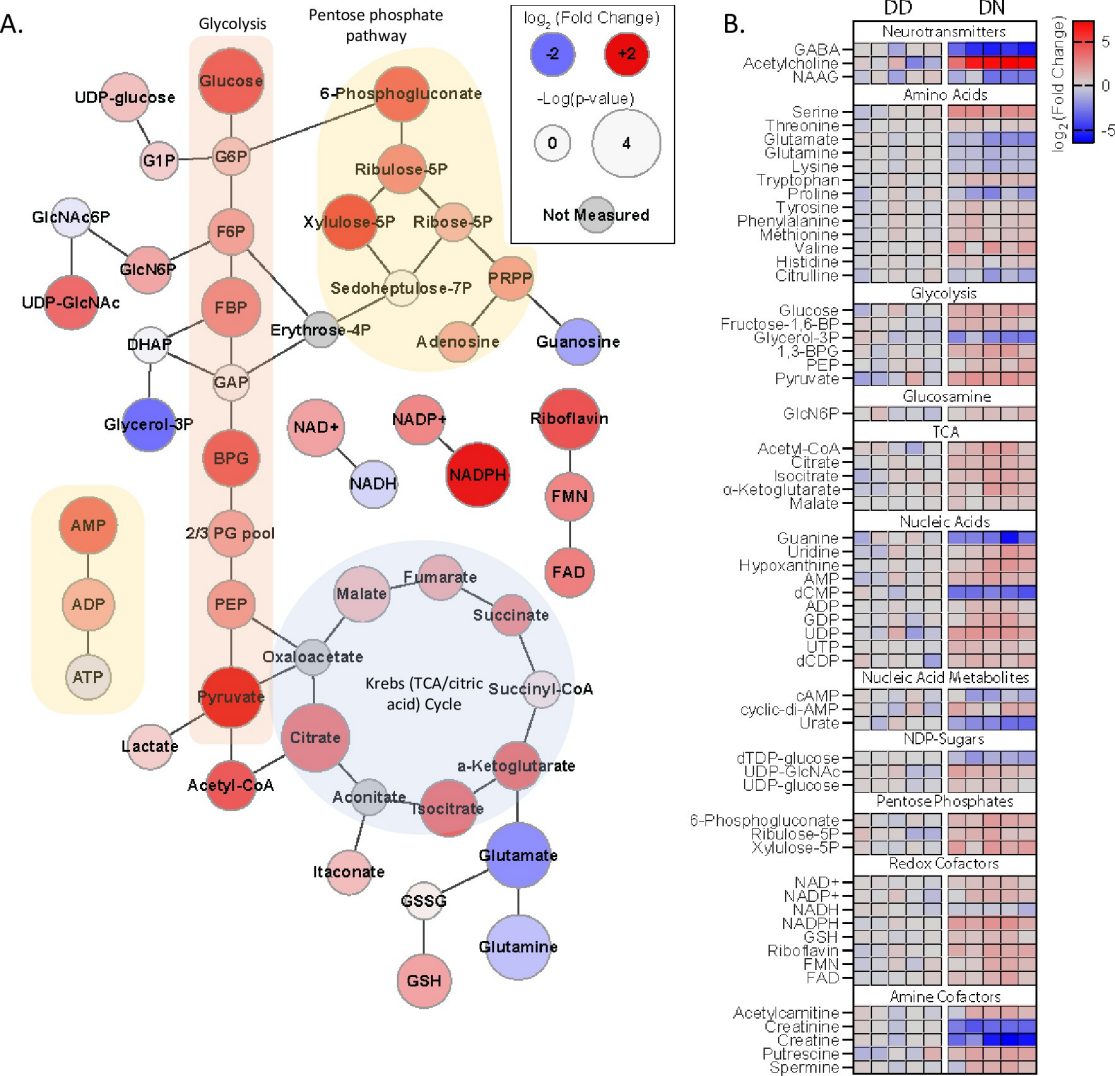
Altered metabolites are associated with the D178 mutation. (A) Web diagram of the metabolites, indicating their roles in glycolysis, the pentose phosphate pathway and Krebs cycle, displaying relative changes in the DN compared with the DD (Log_2_ [fold change]; node color) and the -log(p-value) of the changes (node size). Raw data are in the **S1 Dataset** Metabolomics source data file (n = 5 organoids). (B) Heatmap showing the Log_2_ (fold change) in the levels of various metabolites in the DN relative to the DD COs, each column represents an individual organoid “n”. All data in this Figure were measured on 5-6-month-old COs using LCMS approaches.

The cytosolic oxidative stress could be linked with altered cellular bioenergetics. To explore this, we assessed the levels of CO metabolites (Fig 4). We found that relative to the DD COs, the DN contained increased glycolysis metabolites including fructose 1,6-bisphosphate (FBP), phosphoenolpyruvate (PEP), and pyruvate, demonstrating that the mutation increased reliance on glycolysis. This was supported by the depleted NADH and the increased NAD+ in the DN COs. Interestingly, Krebs cycle metabolites were also increased, supporting that the DN cells had a substantially higher energy demand, which caused them to become highly metabolically active. Glucose levels were higher in the DN than the DD COs, suggesting that the DN COs restricted the use of glucose and utilized an alternative energy source such as beta-oxidation, or, alternatively, increased gluconeogenesis, possibly in astrocytes, to produce more glucose. Both possibilities were supported by the reduced levels of some essential non-carbohydrate metabolites including grycerol-3P, glutamine, and glutamate, which are substrates for beta-oxidation and *de novo* glucose synthesis. Overall, the mutation appeared to upregulate bioenergetics associated with glycolysis and Kreb cycle, which are primarily glucose-dependent, triggering cells to try and maintain sufficient levels of glucose.

### D178N mutation alters mitochondrial function

**Fig 5:**
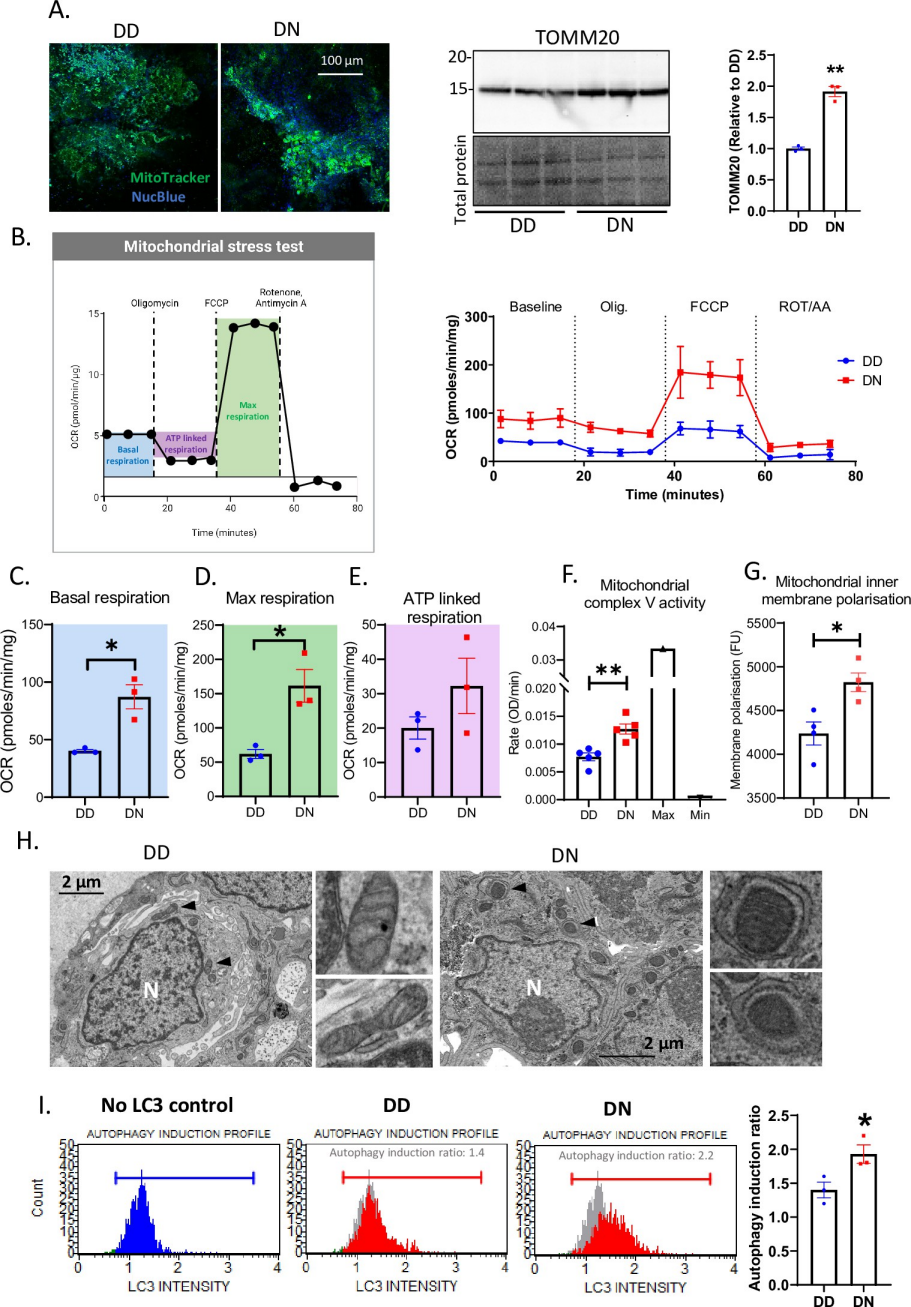
Abnormal mitochondrial activity is associated with increased autophagy/mitophagy in the DN COs. (A) Immunofluorescence images of DD and DN COs labeled with MitoTracker and NucBlue (left panel) and a western blotting analysis of TOMM20 in DD and DN COs (right panel; total protein is the loading control; TOMM). (B-E) Seahorse analysis was used to assess mitochondrial stress by measuring the oxygen consumption rate (OCR) in DD and DN COs at the baseline level, and after sequential treatments with 1 μM Oligomycin, and 2 μM FCCP, 0.5 μM Rotenone/antimycin A, n = 3 (B). (B left panel) A schematic diagram (created with BioRender) displaying how parameters for mitochondrial stress test were extracted from the Seahorse raw data (B right panel). These parameters include the basal respiration (C; blue), maximal respiration (D; green), and ATP linked respiration (E; magenta). (F) Mitochondrial complex V (ATPase) activity measured in the DD and DN COs relative to the maximum activity in the positive control (bovine heart mitochondria) and the minimum activity in bovine heart mitochondria treated with 10 μM oligomycin. (G) The intensity of mitochondrial polarization in DD and DN COs. (H) TEM images of 4-week-old DD and DN organoids with the insets (right panels) showing higher magnification images of mitochondria indicated by the arrowheads. (I) Flow cytometry analysis of autophagy induction in DD and DN COs by detecting cells containing immuno-labeled LC3, with the left panels showing representative profiles (Blue profile shows the no autophagy induction control and this is indicated in grey behind the DD and DN autophagy profiles in red) and the right panel shows autophagy induction ratio. (C-G, I) The average readout in the DN was compared to the DD by Welch’s t test. Each dot is an “n” representing an organoid. Data, unless otherwise stated, were from 5-6-month-old COs and are presented as mean ±SEM. * p<0.05, ** p<0.01.

Changes in the Krebs cycle, as well as the increased mitochondrial localization at the neurites suggests mitochondria may also be compromised in the DN COs, despite the lack of mitochondrial anti-oxidant response. Here we determined if the mutation affects mitochondria. Labeling live mitochondria with MitoTracker showed active mitochondria in both DD and DN COs (Fig 5A two left panels) with the DN harboring more mitochondrial marker TOMM20 (Fig 5A right two panels), suggesting more mitochondria in the mutant COs. The mitochondrial oxygen consumption rate (OCR), gauging the efficacy of the oxidative phosphorylation, was measured at the baseline level, and after sequential treatments with Oligomycin, FCCP, and Rotenone/Antimycin A to modify the oxidative phosphorylation by inhibiting the complex 5, disrupting the proton gradient, and shutting down the mitochondrial function through inhibiting complexes 1 and 3, respectively (Fig 5B). From these measurements, certain parameters can be calculated including basal mitochondrial respiration, maximal respiratory capacity and ATP-linked respiration (Fig 5B left panel). The mitochondria of the DN were more active than the DD COs, with basal and maximal respirations significantly increased (Fig 5C and 5D). While the OCR did not show ATP-linked respiration to be significantly increased (Fig 5E), the overall increased OCR was consistent with the mitochondrial complex V activity being significantly increased and the inner membrane being substantially hyperpolarized in the DN than the DD COs (Fig 5F and 5G). Complex I activity was unaltered in the DN relative to the DD (S4B Fig), suggesting that the mutation does not directly affect every respiratory complex equally. Given that cells typically removed damaged mitochondria by autophagy, we assessed mitophagy by TEM and found more mitochondria undergoing phagocytosis in the DN than the DD COs (Fig 5H). To confirm this visually increased mitophagy in the DN COs, we measured the LC3-dependent autophagy and found significantly higher autophagy in the DN than the DD COs (Fig 5I). This was consistent with a decreased level of uncoupling protein UCP2 in the DN COs (S4C Fig). Thus, an increased energy demand in the DN COs appears to put significant pressure on mitochondrial function, likely resulting in the increased loss of damaged mitochondria by mitophagy coupled with an upregulated mitochondrial biogenesis to maintain viability.

### D178N mutation alters lipid homeostasis

**Fig 6:**
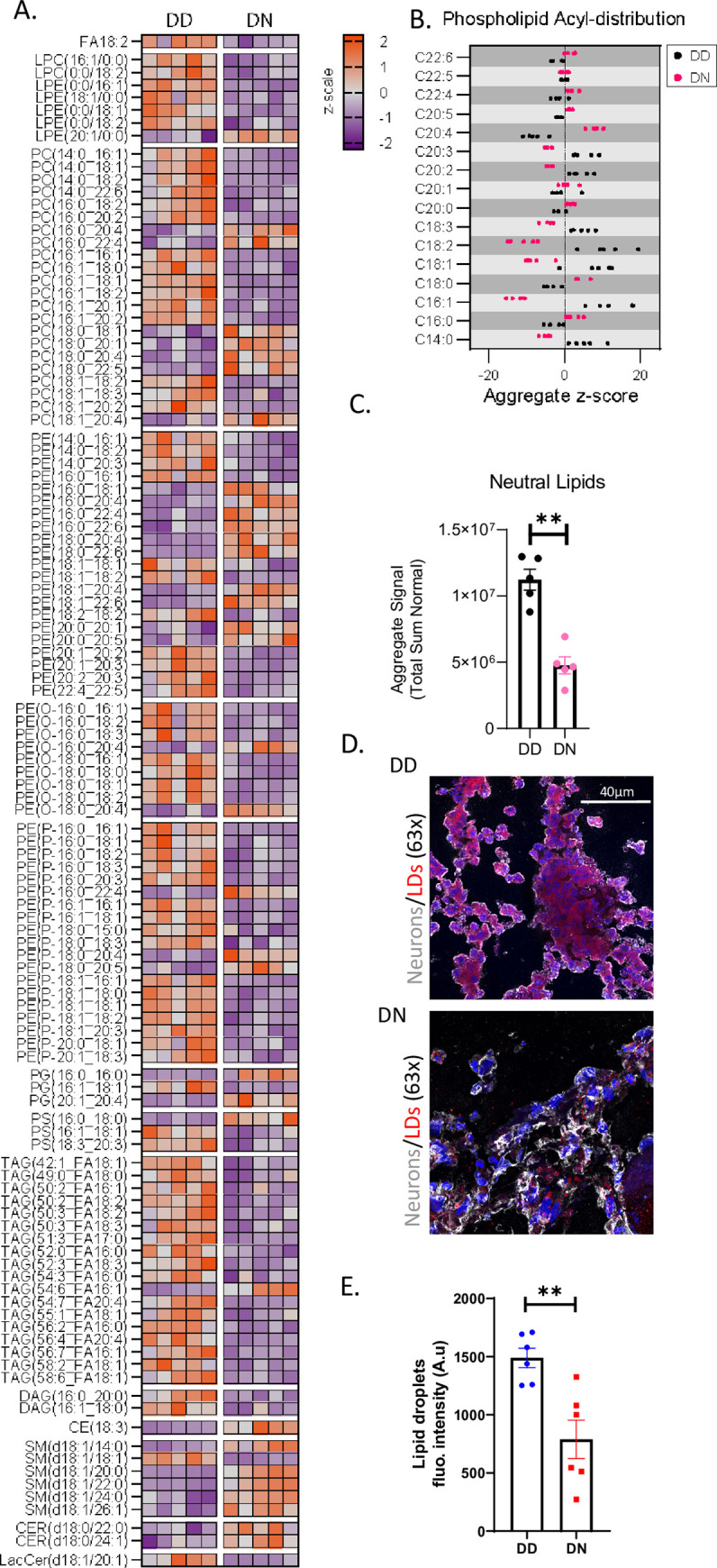
Neutral lipid levels, storage, and metabolism are altered in DN COs. (A) Heatmap displaying the relative levels (z-score) of various species of lipids in five DD and DN COs, each column represents an individual organoid “n”. (B) A scatter plot shows the levels of various neutral lipid species in the DN compared with the DD COs. (C) Total levels of neutral lipids in the DD and DN COs (Welch’s t test). (D) Representative fluorescence images of DD and DN CO sections labeled with MAP2 (for neurons), Nile Red stain (for lipid droplets, LDs), and DAPI, showing LDs in neurons. (E) Quantification of LDs within neurons. The mean LD fluorescence intensity was analyzed by Welch’s t test. Each dot is an “n” representing an organoid. (A, B) Raw data, measured by LCMS, are in the **S2 Dataset** Lipidomic source data file. Data were from 5-6-month-old COs and are presented as mean ±SEM. * p<0.05, ** p<0.001.

An important intermediate in lipid synthesis is acetyl-CoA, which is made in the mitochondria and increased in the DN COs. Since neurodegenerative diseases are associated with altered lipid homeostasis linked to heightened oxidative stress [22, 23], we examined the effect of the D178N mutation on cellular lipid profile via targeted LCMS. Substantial shifts in CO lipid composition were observed including substantial changes in the acyl-chain length across multiple glycerophospholipid and sphingolipid classes that could be associated with a change in cellularity, cellular morphology, or cellular stress responses in the DN COs (Fig 6A and 6B). Additionally, neutral lipids were significantly reduced in the DN relative to the DD COs (Fig 6A–6C). Given that neutral lipids are packaged in lipid droplets (LDs) for further utilization in beta-oxidation and gluconeogenesis [24, 25], we assessed the LDs in the COs and found significantly lower LDs in the DN COs relative to the controls (Fig 6D and 6E). Given that in the process of beta oxidation, mitochondria are recruited toward the LDs to facilitate the use of neutral lipids [24], we assessed the mitochondria and LD-like compartments localization in astrocytes by TEM. We found that the DD astrocytes appeared to harbor more LD-like compartments than the DN astrocytes, and some mitochondria in both lines were localized near LD-like structures (S5 Fig). Overall, the mutation reduces the total neutral lipids and LDs, possibly by utilizing them for energy through beta oxidation.

### Abnormal phenotypes are dependent on the N178 allele

**Fig 7:**
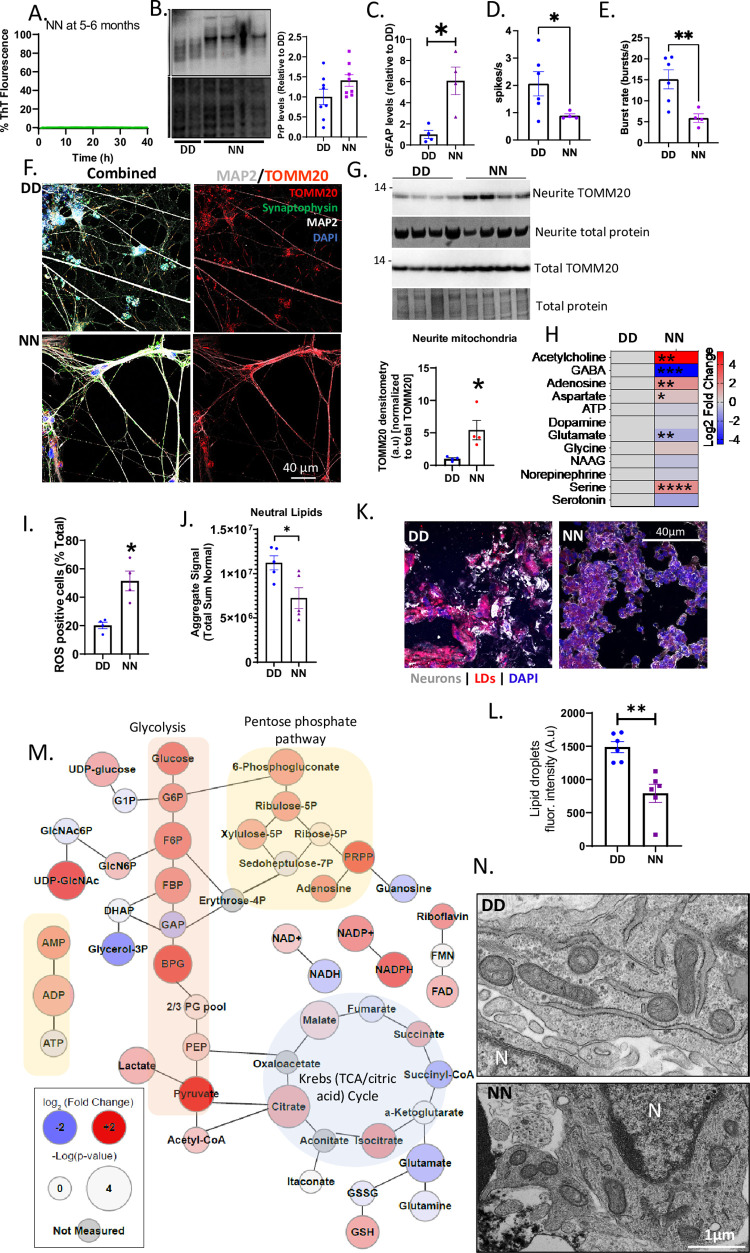
Abnormal phenotypes are dependent on the N178 allele. (A) RT-QUIC analysis of COs with two N178 alleles (NN). (B) A representative western blot for PrP (by 3F4; top panel) with the Coomassie stain for the total protein (bottom panel) and the quantification of PrP relative to the total protein (right panel) (C) Levels of GFAP as detected by western blotting (blot shown in S6A Fig). (D, E) Spike or neuronal firing rate (D) and burst rate (E) of DD versus NN COs. Raw traces of neural oscillations and additional data are in S6B Fig. (F) Representative immunofluorescence images of DD (top panels) and NN (bottom panels) monolayer neurons labeled with TOMM20, synaptophysin, MAP2, and DAPI. The left panels show images with all the stains and the right panels show TOMM20 and MAP2. (G) Western blotting analysis of the neurite TOMM20 levels relative to the total TOMM20 after normalizing to total protein stained by Coomassie, blots are shown above with quantification below. (H) Log_2_ fold changes in the levels of NN neurotransmitters relative to the DD with the degrees of statistical significance determined by a Multiple Student’s t test, n = 5. Raw data are in the **S1 Dataset** Metabolomics source data file. (I) ROS levels in the DD and NN COs measured by flow cytometry. Representative plots are in S6E Fig. (J) The total levels of neutral lipids in the DD versus NN. Additional lipid data are shown in S6F Fig. (K) Representative fluorescence images of DD (left) and NN (right) COs labeled with MAP2 (for neurons), Nile Red stain (for lipid droplets, LDs), and DAPI. (L) Quantification of LDs. (M) Web diagram of the metabolites displaying relative changes in the NN COs compared with the DD (Log_2_ fold change; node color) and the -log(p-value) of the changes (node size), n = 5. Raw data are in the **S1 Dataset** Metabolomics source data file. (N) TEM images of mitochondria in DD and NN COs. (C-E, G, I, J, L) Mean readouts were compared between DD and NN by Welch’s t test. Each dot is an “n” representing an organoid. Data were from 5-6-month-old COs and are presented as ±SEM. * p<0.05, ** p<0.01, *** p<0.001, ****p<0.0001.

Finally, we questioned how much of the phenotypic change in the DN COs is related to the N178 allele versus interaction between the alleles. For this we generated iPSCs harboring two N178 alleles (NN) and examined COs differentiated from these for similarities or differences to the phenotypes exhibited by the DN COs. We observed mostly similar abnormal phenotypes in the NN COs to those seen in the DN when compared with the DD controls (Fig 7). The NN COs showed no prion seeding activity, harbored no insoluble pathogenic prions, and predominantly expressed di-glycosylated PrP as compared with the DD (Figs 7A, 7B, S2A and S2B). However, unlike the DN COs, PrP protein levels were not significantly higher than the control DD COs (Fig 7B). The NN COs also lacked tau and alpha-synuclein seeding activity and harbored more amyloid precursor protein cleavage than the DD controls (S2C, S2D and S2E Fig). Like their DN counterparts, the NN COs demonstrated a heightened astrogliosis (Fig 7C) associated with reduced neuronal network communication (Fig 7D and 7E), high neurite mitochondria (Fig 7F and 7G), changed neurotransmitters (Fig 7H) and oxidative stress (Fig 7I). Lipid composition, including neutral lipids (Figs 7J and S6F) and LDs (Fig 7K and 7L), was accordingly changed. This change was consistent with the NN astrocytes recruiting mitochondria toward LD-like structures, possibly to use neutral lipids for beta oxidation (S6 Fig). Like the DN COs, the NN COs exhibited substantial changes in glycolysis and Krebs cycle intermediates (Fig 7M). One notable difference was observed between the DN and NN COs; while the TEM analysis showed increased mitophagy in the DN COs, the NN COs did not have such a phenotype, thus revealing that the D allele may contribute to this cellular response (Fig 7N). These data indicate that the N allele is the cause of most of the observed phenotype within the DN COs with the exception of heightened mitophagy, which appears to require the presence of the D allele.

## Discussion

Herein, we aimed to investigate the FFI-causing *PRNP* D178N mutation in a human cerebral organoid model. In similarity with previous studies looking at *PRNP* mutations E200K and Y218N (responsible for cases of hereditary CJD and GSS respectively) in human neuronal tissue backgrounds [16, 26], the mutation did not spontaneously cause prion disease. However, the COs recapitulated one hallmark of prion disease, astrogliosis. The increased number and activation of astrocytes was associated with increased PrP detection and fragmentation, and a cytosolic and extracellular response to oxidative stress. Dysfunction of neuronal network communication was related to altered neurotransmitter biosynthesis and abnormal levels and physiology of mitochondria. This correlated with evidence of damaged mitochondria that appeared to trigger an increase in mitophagy and autophagy. Further shifts in energy production intermediates were substantial, with a loss of neutral lipids and lipid droplets.

Increased oxidative stress has been commonly reported in both human brain tissue postmortem and animal models of prion diseases, including FFI [12, 27, 28]. The changes observed affecting PrP itself also indicated cellular stress. Increased PrP expression and increased cleavage, especially C2 cleavage, have been associated with response to oxidative stress [29–33]. Astrogliosis is associated with oxidative stress and lipid peroxidation product accumulation in astrocytes in both CJD and mouse models of prion disease [34]. Oxidative damage is linked to disrupted glucose metabolism in Alzheimer’s disease and prion disease [35, 36]. Enhanced oxidative stress and altered glucose metabolism are often accompanied by mitochondrial dysfunction [37]. Likely the increased energy demand and production of bi-products is contributing to an ongoing cellular stress.

PET imaging of FFI patients shows reduced glucose utilization, especially within the thalamus where the disease is thought to begin [38, 39]. The severity of the hypometabolism is influenced by codon 129 genotype, with patients heterozygous 129MV (M is always cis with the 178N mutation) showing more widespread hypometabolism but a significantly longer disease duration (35 +/- 11 months versus 8.5 +/- 1 month in 129MM patients; [40]). In the brains of sCJD patients, glucose metabolism is decreased in cortical regions but less so in basal ganglia or thalamus (more affected brain regions;[41]). This possibly correlates with the energy shifts observed in the current study as, despite the increase in glycolytic and pentose phosphate pathway intermediates, glucose itself remains high. This might indicate that cells are trying to restrict the usage of glucose by switching to other energy pathways. These shifts in energy pathways, and the more widespread dispersion of hypometabolism in the FFI patients, may represent a cellular compensation for cells undergoing most stress. Indeed, astrocyte networks have been shown to have the capacity to redistribute resources from unstressed to stressed tissue over distances of mm [42].

A shift in organoid metabolism accompanying the astrogliosis is logical as astrocytes and neurons preferentially utilize different metabolism pathways. Neurons demonstrate higher oxidative metabolism and have little capacity to use glycolysis [43, 44]. Instead, neuronal glucose is metabolized through the pentose phosphate pathway, which helps maintain antioxidant balance through production of NADPH [43]. Astrocytes are highly glycolytic with a preference for production of lactate rather than pyruvate for entry into the Krebs cycle. This lactate can then be released for up-take by neurons and use as an energy substrate that bypasses glycolysis altogether. The shifts observed in the metabolite profile suggests the astrocytes could be contributing increased glycolysis and lactate, but sizable increases in reliance on the pentose phosphate pathway and Kreb’s cycle also possibly indicate changes in neuronal metabolism. Further examination of the cellular subsets will be required to determine if such changes are indeed happening as our data on whole organoids cannot differentiate cellular contributions.

Astrocytes are also critically involved in the balance of glutamate and GABA [45]. The metabolomic analysis of energy intermediates demonstrates reduced glutamate and glutamine, likely resulting in the observed reduction in GABA through a blockage of synthesis. As the balance of GABA and glutamate heavily involves astrocyte up-take, the Krebs cycle and replenishment of the glutamate pool requires pyruvate carboxylase, an enzyme predominantly expressed in astrocytes [46, 47], this likely suggests that the astrogliosis in FFI prion disease is associated with dysfunctional homeostasis within the glutamate and GABA systems. Likewise, the increase in acetyl-CoA, the precursor for acetylcholine, likely correlates with the increased detection of the latter, as the rate of acetylcholine synthesis is not only dependent upon the activity of choline acetyltransferase, but also on the concentration of its substrates [48]. These are interesting observations in the context of FFI as forebrain cholinergic neurons contribute to wakefulness; they are active when awake and during rapid eye movement (REM) sleep but inactive during non-REM sleep [49, 50]. Subsets of GABAergic neurons are activated in non-REM sleep and thought to play a role in cortical deactivation [50]. Thus, the two systems balance each other. If during FFI GABAergic function is reduced with increased cholinergic function, then this could cause a push toward a constantly wakeful state resulting in the disturbed sleep patterns. Possibly pharmacological maintenance of balance of these systems could be used as a strategy to delay onset of symptoms or alleviate some symptomology in early FFI and, accordingly, may warrant further investigation.

Shifts in lipid profiles are also quite expected given the changes in available metabolites and mitochondrial function. The observed changes in acyl-chain length across both glycerolipids and sphingolipids may be a neuroprotective mechanism to combat high ROS and general cellular stress [51]. In addition, as the glucose levels remained higher in the DN COs than the DD control, the shift in lipids suggests they may constitute an alternative energy source, independent of glucose, resulting in the increased catabolism of lipid droplets. This suggests the cells may be switching their energy source to maintain their viability under stress.

Increased autophagosomes and autophagolysosomes have been previously observed in FFI mouse brain tissue by EM [5]. This model and another FFI mouse model, developed by a knockin of the human PrP epitopes (3F4) and the mouse equivalent of the mutation (D177N), have demonstrated sleep cycle abnormality, consistent with the disease in humans [4]. Both models showed less un-glycosylated PrP similar to the PrP banding profiles in our organoid model and in terminally ill FFI brain tissues [10]. This was consistent with the DN COs harboring increased levels of UDP-Glucose and UDP-GlcNAc, the primary precursors for protein glycosylation, indicating that the glycosylation pathways are upregulated in the mutant. Both mouse models also produced biochemically abnormal PrP; however only the 3F4 knockin model produced PrP that was transmissible to mice harboring the normal 3F4 epitopes, but not to wild-type mice. These pathogenic properties of PrP are absent in our FFI organoid model. Together, the findings from the mouse and organoid models of FFI suggest that at least some FFI pathological changes do not depend on the ability of PrP to propagate into its misfolded conformers or transmitting species.

One reason that aging-related neurodegeneration may not be manifesting in COs, is the inherent limitation that they more closely resemble the neonatal cerebral cortex than adult [52]. Despite the lack of disease pathology, the observed abnormal phenotypes reveal that the mutation affects the cerebral cortex as early as the neonatal stage of life. However, there has not been a report of any clinical abnormality in neonates who were later identified to be D178N carriers, implying that the abnormal molecular and cellular phenotypes we observed are not severe enough to manifest any clinical phenotypes. Therefore, we speculate that the observed changes are associated with chronic dysfunctions that lead to the disease onset later in life. This is consistent with various reports on cerebral organoid models of genetic neurological disorders displaying some neurophysiological deficits that may lead to the disease onset [13, 17, 53]. Future studies will aim to determine if the observed abnormal phenotypes change or become more deleterious when infection is established in the D178N COs.

While to the best of our knowledge a person homozygous for the D178N mutation has never been reported and, therefore, we acknowledge this CO construct is not physiologically relevant, it does provide additional information about how much contribution is made by the N allele to the observed phenotype. We observed some discrepancies between the DN (heterozygous) and NN (homozygous) COs, with some of the changes appearing milder in the NN than the DN, which suggests that interaction between the allele PrP products may exacerbate the phenotype. As we were only able to obtain one clone for the NN genotype it must also be considered that this effect could be due to clonal drift reducing the severity of the phenotype. However, most of the observed changes were present in the NN COs implicating this allele as primarily responsible, without a dose effect.

## Conclusions

Herein we demonstrated that a cerebral organoid model of FFI harbored no replicating prion disease. Despite the lack of active disease, the mutation enhanced oxidative stress associated with various abnormal phenotypes including; astrogliosis, impaired neuronal network communication, altered bioenergetics, dysfunctional mitochondrial activity, increased mitophagy and autophagy, and altered lipid metabolism. These abnormalities are likely features of asymptomatic FFI that may lead to the disease.

## Methods

### Human ethics statement

The human samples used in this study were obtained from a commercial source (Applied Stem Cell). Thus, the NIH Office of Human Subjects Research Protections (OHSRP) has determined these samples to be exempt from National Institutes of Health (NIH) Institutional Review Board (IRB) review.

#### iPSC cloning

Induced pluripotent stem cells (iPSCs) from an individual with no history of genetic neurological disease were sourced from Applied Stem Cell (ASE-9209). Both PRNP alleles contained aspartic acid (D) at codon 178 and methionine (M) at codon 129. Applied Stem Cell designed the CRISPR-Cas9 cloning strategy, performed the cloning, selection and quality control and provided two clones of each genotype (except for the NN where only one clone was obtained). There were seven potential off-target binding sites identified; all were in non-coding regions with 3 base pair mismatches and therefore deemed unlikely to influence cell phenotypes (S1 Fig). NN iPSCs were cloned directly from the DD cells (not from a sequential cloning using the DN iPSCs).

### Cerebral organoid generation and culture

IPSCs were routinely cultured on low growth factor Matrigel (Roche) in mTeSR1 or mTeSR Plus medium (StemCell Technologies) under the conditions previously described [15]. Organoid differentiation was performed using the cerebral organoid differentiation kit (Stem Cell Technologies), which is based on the original protocol developed by Lancaster and Knoblich [54]. Following differentiation, cultures were maintained on an orbital shaker at 85 rpm in complete maintenance medium (1 × glutamax [ThermoFisher], 1 × penicillin–streptomycin solution [ThermoFisher], 0.5 × non-essential amino acids [Sigma Alrich], 0.5% [v/v] N2 [ThermoFisher], 1% (v/v) B12 with retinoic acid [ThermoFisher], 0.025% (v/v) insulin [Sigma Aldrich], and 0.00035% (v/v) 2-Merceptoethanol [Sigma Aldrich] in 1:1 Neurobasal:DME-F12 medium [ThermoFisher]), under standard incubator conditions (5% CO_2_, 37°C, humidified). For DD and DN differentiations both clones were used to generate cultures. Although not all assays were performed on both clones, where both were used they have been combined in the same analysis. Organoids from at least 3 independent differentiations were used for the studies herein and differentiations of organoids from each genotype were started at the same time to avoid media or incubator drift.

### Forebrain neuron differentiation and culture

iPSCs were differentiated into forebrain neurons using the StemCell Technologies single cell passage protocol. Initial growth was in STEMdiff SMADi neural induction kit media (StemCell Technologies) up to passage 3 then the medium was changed to the STEMdiff Forebrain Neuron differentiation and subsequently STEMdiff Forebrain Neuron Maturation kits (StemCell Technologies) as per the manufacturer’s instructions. Forebrain neurons were maintained in maturation medium until assay. For electrophysiological function assays, forebrain neurons were plated at a density of 1 ×10^4^ cells/well (~3 ×10^4^ cells/cm^2^) in low growth factor matrigel coated 24-well MEA plates with PEDOT electrodes on glass (Multichannel systems) at the time of passage into maturation medium. Readings were taken after 4 weeks of maturation.

### Astrocyte differentiation and culture

Neural progenitor cells (NPCs) were differentiated from iPSCs using STEMdiff SMADi Neural induction medium (StemCell Technologies). Astrocytes were then differentiated from the NPCs as described by Tcw and colleagues [55] using astrocyte medium (ScienCell). At passage 4, approximately 4 weeks of culture in astrocyte medium, cultures were magnetically sorted to remove remaining GLAST negative cells using MACS human/mouse GLAST magnetic beads (Miltenyi) as per the manufacturer’s instructions. Cells were used for experiments within two passages of GLAST sorting.

### Neurite culture and analysis

To induce neurites and axons to grow through a well insert membrane (with somas remaining on the other side; Fig 2J), the underside of 0.4 μm pore 24-well inserts (Millipore) was coated with low growth factor matrigel. Differentiated neurons were plated into the well insert at a density of 1 × 10^5^ cells/insert in neuronal maturation medium (STEMdiff Forebrain Neuron Maturation kit) and incubated for 7 days with media changes every 3 days. To analyze the neurite contents, neurons cultured on inserts were fixed with 100% (v/v) ice cold methanol for 10 minutes and washed with 1× PBS. The neuronal somas were scraped off the top of the inserts, leaving only the neurites attached to the bottom of the inserts. The inserts containing the neurites were placed on 50 μL droplets of RIPA buffer (Sigma; containing 1× protease inhibitor) for 30 minutes to lyse the neurites. The lysates were collected to analyze the neurite mitochondria by western blot.

### Neuro-electrophysiology

As described previously [17], we used Multi-electrode arrays (MEA; embedded with 60 titanium platinum microelectrodes) in a MEA2100-System with an integrated amplifier (Multichannel Systems) to record the neuro-electrophysiology of COs. The recording was conducted in BrainPhys media (StemCell Technologies) at 32°C. The COs were held firmly to the electrodes by harp slice grids (Multichannel Systems). The neuronal population firings (spikes) were detected as those greater than 4 standard deviations of the mean (calculated in the first 30 sec) after filtering with a High-pass filter (300–2500 Hz) using McRack software (Multichannel Systems). The population neuronal burst (>4 spikes/100ms) and its periodicity were calculated by autocorrelation using MEAnlyzer (MATLAB toolbox) [56]. The relative oscillatory powers were calculated using MATLAB Signal Processing and Wavelet toolboxes as described previously [17]. Raw signals were filtered by FIR filter, down sampled from 25,000 to 1000 Hz, transformed into time-frequency domain by continuous wavelet, and decomposed into narrow-band frequency ranges (delta: < 4 Hz, theta: 5–8 Hz, alpha: 9–13 Hz, beta: 14–32 Hz, low gamma 33–80 Hz, upper gamma: 100–200 Hz) by inverse wavelet. The relative oscillatory power of each narrowband was estimated by Welch’s method with a window length of 2000 ms and an overlap of 1000 ms.

For the monolayer neurons plated in the multi-well electrodes (described above in the Forebrain neuron differentiation and culture protocol), the raw data were sampled at 20,000 Hz and filtered with a High-Pass filter (300 Hz; 2^nd^ order Butterworth) and a Low-Pass filter (3500 Hz; 4^th^ order Butterworth) using the Multiwell-Screen software (Multichannel Systems). The population neuronal firings (spikes) were detected as those greater than 4 standard deviations of the mean (calculated in the first 10 sec). Neuronal population burst consisted of at least 4 spikes within 100 ms.

### Reactive oxygen species (ROS) assay

This assay was performed as described in the Muse Oxidative Stress Kit (Luminex). The organoids were dissociated using accutase, pelleted by a 5-minute 300×g spin, and resuspended in 1× Assay Buffer into ~1×10^6^ cells/mL. For each reading, 10 μL of the cell sample was added into 190 μL of oxidative stress working solution (prepared by diluting the Muse Oxidative Stress Reagent 1:100 with 1× Assay Buffer and further diluting 1:80 with 1× Assay Buffer) and incubated at 37°C for 30 minutes before analyzing using Guava Muse Cell Analyzer (Luminex). Relative to a negative control that was not treated with the Muse Oxidative Stress working solution (no oxidative stress), cells were gated to be either, M1—cells negative ROS, or, M2—cells positive for ROS.

### MitoPotential assay

This assay was performed as described in the Muse MitoPotential Kit (Luminex). The organoids were dissociated using accutase, pelleted by a 5-minute 300×g spin, and resuspended in 1× Assay Buffer into ~1×10^6^ cells/mL. For each sample, 100μL of resuspended cells was added to 95μL of the Muse MitoPotential working solution (prepared by diluting MitoPotential Dye 1:1000 with 1× Assay Buffer), mixed thoroughly, and incubated for 20 minutes in a 37°C CO2 incubator. The measuring of the MitoPotential intensity was performed using the Guava Muse Cell Analyzer after adding 5 μL of the Muse MitoPotenital 7-AAD.

### Mitochondrial Complex 1 activity assay

The complex 1 activity assay was performed using the Complex 1 Enzyme Activity Microplate Assay Kit (Colorimetric) (Abcam cat. ab109721). COs were lysed in the Detergent (prepared by diluting the stock Detergent 1:10 with 1×PBS) into ~10% (w/v) lysates. The protein concentration was determined by BCA assay and corrected between samples. The lysates (200μL/sample/well) were added to the microplate and incubated at room temperature for 3 hours. Each well was washed 3 times with 1× Wash buffer solution (300 μL/wash) and filled with 200 μL assay solution (containing 1× Dilution buffer, 1×NADH and 1× Dye). The complex 1 activity was measured in ClarioStar plate reader (BMG) at 450 nm wavelength for 30 minutes (at room temperature) in 30 sec intervals with a shake between readings.

### Mitochondrial Complex 5 activity assay

The complex 5 activity assay was performed using the MitoTox Complex V OXPHOS Activity Assay Kit (Abcam cat. ab109907). The positive control was the bovine heart mitochondria (BHM; demonstrating the max complex 5 activity) provided by the kit, and the negative control was the BHM treated with 20 μM oligomycin (demonstrating the minimum activity). COs were lysed into 20% (w/v 1×PBS) and 40 μL of each sample was for the assay as described in the protocol. The complex 5 activity was measured in ClarioStar plate reader (BMG) at 350 nm wavelength for 2 hours at 30°C protected from light.

### Autophagy assay

This assay was performed according to the Muse Autophagy Assay kit (Luminex; cat. CF2000930). Briefly, COs were incubated in the Reagent A (1:10,000 dilution) for 6 hours in a 37°C CO_2_ incubator to induce autophagy, dissociated using accutase, and labeled with Anti-LC3 AlexaFluor 555 antibody for 30 minutes before using the Guava Muse Cell Analyzer to count the LC3 positive cells. The negative control CO was not treated with the Reagent A.

### Presto blue (cell metabolism)

Cell metabolism was measured by PrestoBlue Cell Viability Reagent (Invitrogen Cat. A13262) as described previously [15]. COs were incubated in PrestoBlue solution (1:10 dilution in complete maintenance media) for 15 minutes in a CO_2_ incubator. The fluorescence of the PrestoBlue solution was measured at 590 nm in ClarioStar plate reader (BMG).

### Calcium flux assay

COs intracellular calcium level was measured by Fluo-4 Direct Calcium Kit (ThermoFisher; F10471) as described previously [17]. COs were incubated in 50% (v/v, in A+ media) 2× Fluo-4 Direct calcium reagent at 37°C without CO_2_ for 1hr (in ClarioStar plate reader, BMG), while the fluorescence of the reagent was read at 494 nm excitation and 516 nm emission in a circular pattern every minute.

### Seahorse assay

Mitochondrial function was assessed using the SeaHorse XFp analyzer (Agilent) and Mitostress Assay kit as described in the manufacturer’s protocol. Seahorse DMEM media (Agilent) was made up to the following specifications throughout; 25 mM glucose, 1 mM pyruvate, 2 mM glutamine (Agilent). FCCP calibrations were performed and the optimal concentration suitable for the COs was 2 μM. COs were dissociated using accutase and adhered to the bottom of Seahorse plate by Matrigel. A BCA assay was performed on the cells following the Seahorse assay, and the mitochondrial function was normalized to total protein. Results were analyzed using Agilent’s Wave software.

### Metabolite and lipid sample preparation

For all LCMS methods LCMS grade solvents were used. CO samples were collected in 0.5 mL of ice-cold methanol. Samples were homogenized and the homogenate recovered. To each sample 0.5 mL of water and 0.5 mL of chloroform was added. Samples were agitated for 20 minutes at 4°C and subsequently centrifuged at 16,000 ×g for 20 min to induce layering. 400 μL of the top (aqueous) and bottom (organic) layer were collected. The aqueous layer was diluted 5x in 1:1 methanol:water for LCMS injection. The organic layer was dried in a Savant SpeedVac SPD130 (Thermo Scientific) and resuspended in 1 mL of 5 μg/mL butylated hydroxytoluene in 6:1 isopropanol:methanol. Organic fractions were further diluted 5x in the same solvent and prepared for injection.

### Liquid chromatography tandem mass spectrometry

Tributylamine and all synthetic molecular references were purchased from Millipore Sigma. LCMS grade water, methanol, isopropanol and acetic acid were purchased through Fisher Scientific.

Aqueous metabolites were analyzed using a previously established ion pairing method with modification with the addition of previously established neurotransmitter signatures [17, 57, 58]. Aqueous fraction samples were separated using a Sciex ExionLC AC system and measured using a Sciex 5500 QTRAP mass spectrometer. Quality control samples were injected regularly to monitor for signal stability. Peaks were resolved with a Waters Atlantis T3 column (100 Å, 3 μm, 3 mm X 100 mm) using a binary gradient from 5 mM tributylamine, 5 mM acetic acid in 2% isopropanol, 5% methanol, 93% water (v/v) to 100% isopropanol over 15 minutes. Each metabolite was identified using at least two distinct multiple reaction monitoring (MRM) signals and a defined retention time.

Bulk lipids were analyzed as previously described [58]. A Shimadzu Nexera LC-20ADXR was used for chromatography. A Water XBridge Amide column (3.5 μm, 3 mm X 100 mm) with a 12-minute binary gradient from 100% 5 mM ammonium acetate, 5% water in acetonitrile apparent pH 8.4 to 95% 5 mM ammonium acetate, 50% water in acetonitrile apparent pH 8.0 was used to separate organic fraction samples. Lipids were detected using a Sciex 6500+ QTRAP mass spectrometer with polarity flipping. Lipids were detected using scheduled MRMs.

All signals were integrated using MultiQuant Software 3.0.3. Signals with greater than 50% missing values were discarded and remaining missing values were replaced with the lowest registered signal value. All signals with a QC coefficient of variance greater than 30% were discarded. Metabolites with multiple MRMs were quantified with the higher signal to noise MRM. Filtered datasets were total sum normalized prior to analysis. Single and multi-variate analysis was performed in MarkerView Software 1.3.1. All univariate comparisons were subjected to a Benjamini-Hochberg cut-off for false discovery as indicated.

### Transmission Electron Microscopy (TEM)

COs and monolayer cultures (cultured on Thermanox coverslips) were fixed at 4°C with 2.5% glutaraldehyde/2% paraformaldehyde in 0.1 M sodium cacodylate buffer, pH 7.4. A solution containing 0.5% osmium tetroxide/0.8% potassium ferricyanide was used to post-fix COs for 1 hr and monolayer cultures for 45 mins. Specimens were stained for 1hr. in 1% tannic acid. COs were post-fixed again under vacuum in 2% osmium tetroxide in 0.1 M sodium cacodylate buffer and stained for 1 hr. under vacuum in 1% uranyl acetate. Specimens were dehydrated overnight in 75% ethanol, followed by a graded ethanol series the next day, and embedded in Spurr’s resin. Thin sections between 70–90 nm were cut with an RMC MT-7000 ultramicrotome and viewed at 80 kV on a Hitachi 7800 transmission electron microscope and digital images were acquired.

### Prion RT-QuIC assay

Real-time quaking-induced conversion (RT-QuIC) assays were performed similarly to those reported previously [25]. Briefly, the RT-QuIC reaction mix contained a final concentration of 10 mM phosphate buffer (pH 7.4), 300 mM NaCl, 0.1 mg/mL bank vole recombinant PrP23–230 M109I [7], 10 μM thioflavin T (ThT), and 1 mM ethylenediaminetetraacetic acid tetrasodium salt (EDTA). COs were homogenized by motorized pestle to 10% (w/v) in PBS and cleared with a 2000× g 2 min centrifugation. CO homogenates were serially diluted in SDS/PBS/N2 solution, using 0.05% (v/v) SDS for a final SDS concentration in the reaction mix of 0.001% (w/v). A volume of 98 μL of reaction mix was loaded into a black 96-well plate with a clear bottom (Nunc), and reaction mixtures were seeded with 2 μL of a 103-fold dilution of CO homogenate for a final reaction volume of 100 μL. Reactions were in quadruplicate for each sample. Plates were sealed (Nalgene Nunc International sealer) and incubated in a BMG FLUOstar Omega plate reader at 42°C for 40 h with cycles of 60 s of shaking (700 rpm, double-orbital) and 60 s of rest throughout the incubation. ThT fluorescence measurements (excitation, 450 ± 10 nm; emission, 480 ± 10 nm [bottom read]) were taken every 45 min.

### α-synuclein RT-QuIC analysis

The K23Q α-synuclein substrate was purified, and the assay was run as previously described [17, 59]. Brain and organoids were homogenized to 10% in PBS and cleared with a brief 2000×g 2 min centrifugation. Two microliters of the specified organoid dilution were added to 98 μL of reaction mix containing final concentrations of 40 mM phosphate buffer (pH 8.0), 170 mM NaCl, 0.1 mg/ml of K23Q α-synuclein substrate, and 10 μM ThT. Each organoid dilution was assessed in quadruplicate in a 96-well optically clear bottom plate (NUNC). Each well was preloaded with six glass beads (0.8 mm in diameter, OPS Diagnostics). Plates were sealed, placed in an Omega FLUOStar plate reader at 42°C and subjected to cycles of 1 min of double orbital shaking at 400 rpm and 1 minute of rest for 40 h. ThT fluorescence reads (450 excitation, 480 emission) were taken every 45 min. Samples were considered positive for seeding activity if the fluorescent signal for >25% of the replicate wells exceeded 10% of the maximum value on the plate prior to 40 h. Data was plotted using Graphpad Prism.

### Tau RT-QuIC analysis

The K12CFh substrate was purified and the assay was performed as previously described [17, 60]. Briefly, brain and organoid homogenates (10% w/v in PBS) were serially diluted in tenfold steps in a dilution buffer containing 0.53% tau-free mouse brain homogenate (KO; tauGFP from Jackson Laboratories) and 1 × N2 Supplement (Gibco) in 10 mM HEPES. Two microliters of the specified organoid dilution were added to 48 μL of reaction mix containing 0.1mg/ml K12CFh substrate, 40 mM HEPES (pH 7.4), 400 mM NaF (buffered in 10 mM HEPES, pH 7.4), 40 μM heparin, and 10 μM thioflavin T. Each organoid dilution was assessed in quadruplicate in a 384-well optically clear bottom plate (NUNC). Plates were sealed, placed in an Omega FLUOStar plate reader at 42°C and subjected to cycles of 1 min of orbital shaking at 500 rpm and 1 minute of rest for 50 h. ThT fluorescence reads (450 excitation, 480 emission) were taken every 15 min. Data was plotted using Graphpad Prism.

### Sarkosyl insolubility assay

As described previously[16], lysates (20 μL of 10% w/v in RIPA lysis buffer) were treated with 300 μL of 10% (w/v) sarkosyl for 1 h at RT with constant agitation at 1400 rpm, diluted with 2680 μL of H-Buffer, and centrifuged at 100,000× g for 1 h at 4°C. The pellets were resuspended in 1× sample buffer for Western blot analysis.

### Proteinase-K (PK) digest

As described previously [17], 10% (w/v) lysates containing 1% (v/v) sarkosyl were digested with 10 ug/ml PK at 37°C for 1hr, and boiled for 5 minutes in 1x sample buffer (Invitrogen) containing 6% (v/v) 2-Mecaptoethanol to denature the proteins and inactivate PK. Samples were analyzed for PrP levels by western blotting.

### PNGaseF and EndoH digest

The PNGaseF digest was performed using the New England BioLabs PNGaseF kit (P0704S), and the Endo H digest was performed using a kit from Promega (Cat. V4875). About 9 μl lysate (20% w/v in 1× PBS) was denatured by boiling for 10 minutes in 1× denaturing buffer (by adding 1 μl of the 10× Denaturing Buffer). The other components of the reaction were added to the denatured samples depending on the digest protocol. For the PNGaseF digest, 2 μL of the GlycoBuffer 2 (10×), 2 μL of 10% NP-40, 5 μL of dH2O, and 1 μL of PNGase F (500K U/ml) were added to make a 20 μL total reaction volume. For the Endo H digest, 2 μL of the 10× Endo H reaction buffer, 3 μL of Endo H (50K units), and 5 μL dH2O were added to make a 20 μL reaction volume. Both PNGaseF and EndoH reactions were incubated at 37°C for 18hrs before boiling for 5 mins in 1× sample buffer for western blotting analysis.

### Immunofluorescence

As described previously [17], COs were fixed in 10% (v/v) formalin for 24 hrs at room temperature, washed with 1×PBS, incubated in 10–20% sucrose for 24 hrs at room temperature, coated with a blue dye, embedded in OCT, and frozen at 20°C for 1–2 hrs, and sectioned into 10μm thick slices using a cryostat (LEICA CM 3050 S). The slices were incubated for 1 hr at room temperature in a blocking solution containing 0.3 M glycine (formalin quenching solution), 0.1% Triton X-100 (permeabilizing solution), and 5% (w/v) BSA (blocking solution) to quench formalin, permeabilize cells, and block nonspecific bindings. The protein of interest was labeled with primary antibody (in blocking solution) by incubating at 4°C for overnight and the appropriate secondary antibody by incubating at room temperature for 1 hr (see S1 Table for antibody information). The cell nuclei were labeled by incubating in 299 nM DAPI for 5 mins at room temperature. Images were taken using an EVOS-FL-auto light microscope (Invitrogen) or Confocal microscope (Zeiss laser scanning LSM 880 microscope driven by ZEN v.2.3 software). For live cell imaging, mitochondrial were labeled by incubating in 200 nM MitoTracker solution (ThermoFischer Scientific; in a phenol red negative DMEM) for 45 minutes in a 37°C incubator with 5% CO_2_, and the cell nuclei were labeled with Nucblue stain (Invitrogen) for 5 minutes at room temperature. The stained live COs were washed and imaged at 10× magnification in the Confocal microscope. For monolayer neurons fixed in 10% formalin (for 15 minutes), cells were washed with 1×PBS, treated with 200 mM glycine for 5 mins (to quench formalin), permeabilized with 0.1% (v/v) Triton-X-100 for 10 minutes, washed with 1× PBS, and blocked with 10% (v/v) fetal bovine serum and 0.5% (w/v) bovine serum albumin. Cells were then washed with 1× PBS, incubated with primary antibody (S1 Table) in 1% (v/v) fetal bovine serum and 0.5% (w/v) bovine serum albumin for overnight at 4°C, washed repeatedly with 1×PBS, incubated in the appropriate secondary antibody for 1hr at room temperature, washed with 1×PBS containing 290nM DAPI, and cured with ProLong-Gold Antifade Mounting media (Invitrogen) for 24 hrs at room temperature before imaging.

### Western blot

Lysates were denatured by boiling for 5 minutes in 1× sample buffer containing ~6 (v/v) beta-Mercaptoethanol. Denatured lysates were resolved (~27 μL lysate/well) in Bolt 4–12% Bis-Tris gels (Invitrogen) and transferred to PVDF membrane (Millipore). The marker was either the SeeBlue or the Novex Sharp Pre-Stained Protein Standard (Life Technologies). The membrane was blocked with the EveryBlot Blocking Buffer (Biorad) for 10 minutes and incubated in the primary antibody (for 1hr at room temperature or overnight at 4°C) and in the appropriate secondary antibody for 1hr at room temperature (see S1 Table for the antibody information and dilution). The protein bands were visualized using ECL Select (Amersham), SuperSignal West Femto Maximum Sensitivity Substrate, or SuperSignal West Atto Ultimate Sensitivity Chemiluminescent Substrate (Thermo Scientific) and imaged by iBright imaging system (Invitrogen). Full blot images are shown in S1 Fig.

### Data analyses

Western blotting quantification was performed using ImageJ. All statistical analyses were performed using GraphPad Prism 8. For experiments with organoids and monolayer neurons, each “n” represents a biological repeat, which is an individual organoid or a chamber of neurons respectively. Each dot on a dot plot is an individual “n”. The statistical analysis method used for each analysis is listed in the Fig legends.

## Supporting information

S1 Fig Selected gRNA and off target profile. (TIF)

S2 Fig The neurodegenerative disorder-associated proteins prions, α-synuclein, and tau were undetectable in the mutant COs. (A) A western blot analysis of PrP in DD, DN, and NN COs as well as in mouse brain homogenate infected with 22L scrapie (PrP^Sc^) after digesting with proteinase-K (PK). (B) A western blot analysis of PrP in DD, DN, and NN COs as well as in mouse brain homogenate infected with PrP^Sc^ after solubilizing with sarkosyl. (C, D) No evidence for accumulation of disease associated α-synuclein or Tau. RT-QuIC analysis for misfolded α-synuclein (C) or tau (D). Percent positive replicated wells (see Methods) are shown for each dilution. Dilutions are represented as log dilution of the initial brain or organoids tissue. Brain tissue controls are shown as squares while organoid samples are shown as circles. Colored markers indicate dilutions. Dashed line in A indicates the 25% positivity threshold which a sample needs to be above to be considered positive. DLB = dementia with lewy bodies, CBD = corticobasal degeneration, CVD = cerebrovascular dementia, sAD = sporadic Alzheimer’s Disease, KO = Tau knock-out mouse. (E) Western blotting analysis of amyloid precursor protein and amyloid beta using 6E10 antibody. The middle panel is an longer exposure of the blot to maximize the visibility of amyloid beta. The lower panel is a Coomassie stain for total protein. Molecular weight markers (KDa) are listed on the left. Data were from 5-6-month-old COs.
(TIF)

S3 Fig Relative oscillatory power of delta and theta oscillations. (TIF)

S4 Fig Mitochondrial function. (A) Analysis of mitochondrial complex 1 activity (see Methods for details) in the DD and DN COs. The left panel shows the full 30-minute recording. The right panel shows the average of the last 5 minutes. (B) Western blotting for UCP2 in DD and DN organoids (top left panel), the Coomassie stain for the total protein (bottom left panel), and the quantification of the levels of UCP2 after correcting to the total protein (right panel). (A, B) Mean readouts were compared between CO lines by Welch’s t test. Data are presented as ±SEM. * p<0.05 Data were from 5-6-month-old COs and are presented as ±SEM. * p<0.05.
(TIF)

S5 Fig TEM analysis of lipid droplets (LDs)-like microcompartments and their localization with mitochondria in DD, DN, and NN astrocytes. (TIF)

S6 Fig Additional data on the comparison between the NN and the DD COs. (A) Western blot analysis of GFAP levels in DD, DN, and NN COs. (B) Raster plots (bottom panels) and neuronal firing frequency plots (upper panels) of ~5–6 month old DD and NN COs. (C) Representative traces of the delta, theta, and gamma oscillations of the neuronal electrical signaling of DD and NN COs. (D) Relative oscillatory powers of the lower gamma (left panel) and upper gamma (right panel) oscillations in DD and NN COs. Mean oscillatory powers were compared between groups by Welch’s t test. Each dot is an “n” representing an organoid. (E) Representative plots from the analysis of ROS in the DD (upper panel) and NN (lower panel) by flow cytometry. (F) A heatmap shows the levels of various lipids in five DD and five NN COs. Raw data are in the **S2 Dataset** Lipidomic source data file. Data were from 5-6-month-old COs.
(TIF)

S1 Table Antibody information used for Western blotting (WB) and Immunofluorescence (IF) analyses. (TIF)

S1 Dataset Metabolomics source data. (XLSX)

S2 Dataset Lipidomic source data. (XLSX)

S3 Dataset Numerical data contained within figures. (XLSX)
