# Supplementary figures and images for: Altered energy metabolism in Fatal Familial Insomnia cerebral organoids is associated with astrogliosis and neuronal dysfunction
Source: PLoS Genet. 2023 Jan 19;19(1):e1010565. doi: 10.1371/journal.pgen.1010565 (PMC9851538; doi:10.1371/journal.pgen.1010565)

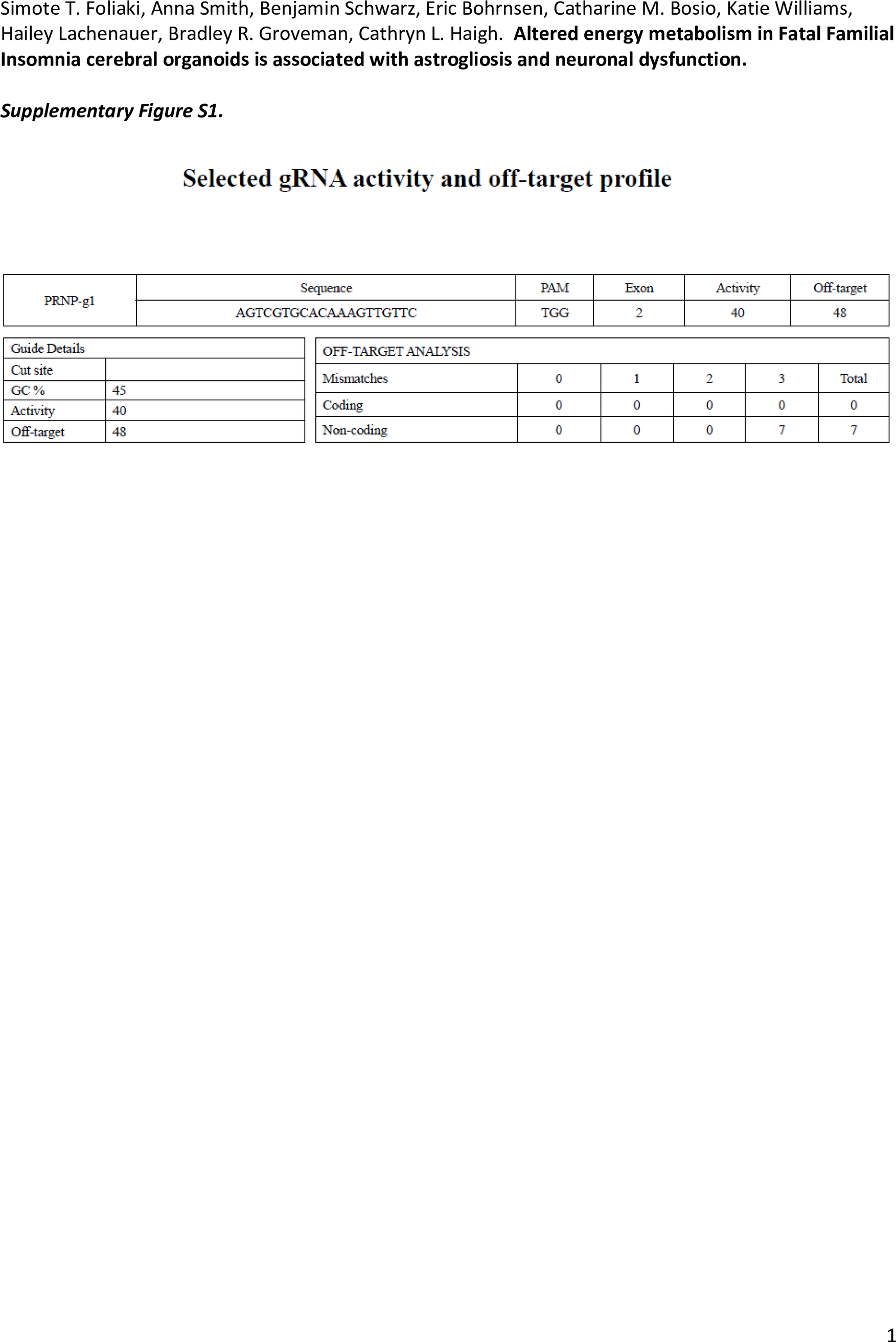

Supplement: S1 Fig — (TIF) [file pgen.1010565.s001.tif]

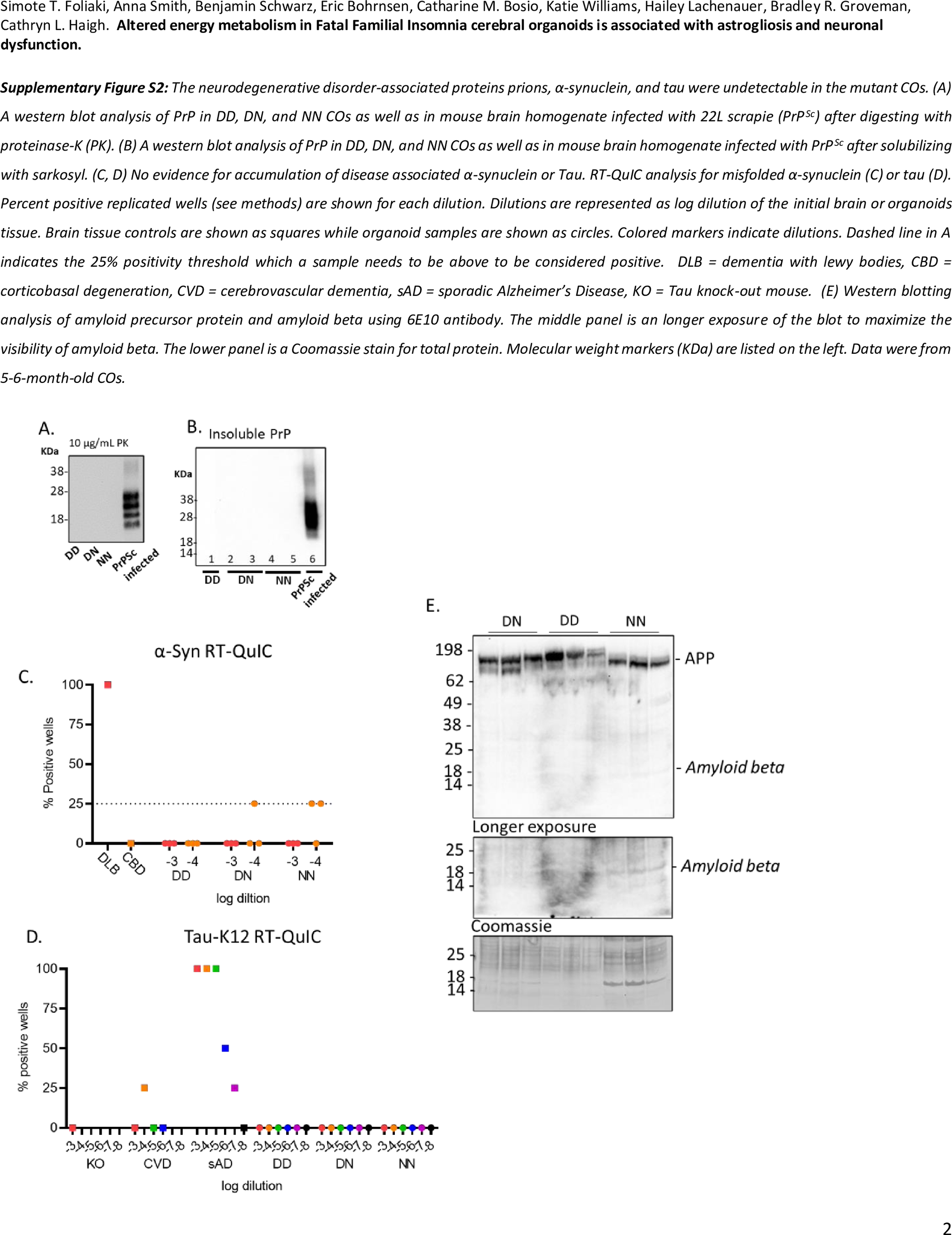

Supplement: S2 Fig — (A) A western blot analysis of PrP in DD, DN, and NN COs as well as in mouse brain homogenate infected with 22L scrapie (PrPSc) after digesting with proteinase-K (PK). (B) A western blot analysis of PrP in DD, DN, and NN COs as well as in mouse brain homogenate infected with PrPSc after solubilizing with sarkosyl. (C, D) No evidence for accumulation of disease associated α-synuclein or Tau. RT-QuIC analysis for misfolded α-synuclein (C) or tau (D). Percent positive replicated wells (see Methods) are shown for each dilution. Dilutions are represented as log dilution of the initial brain or organoids tissue. Brain tissue controls are shown as squares while organoid samples are shown as circles. Colored markers indicate dilutions. Dashed line in A indicates the 25% positivity threshold which a sample needs to be above to be considered positive. DLB = dementia with lewy bodies, CBD = corticobasal degeneration, CVD = cerebrovascular dementia, sAD = sporadic Alzheimer’s Disease, KO = Tau knock-out mouse. (E) Western blotting analysis of amyloid precursor protein and amyloid beta using 6E10 antibody. The middle panel is an longer exposure of the blot to maximize the visibility of amyloid beta. The lower panel is a Coomassie stain for total protein. Molecular weight markers (KDa) are listed on the left. Data were from 5-6-month-old COs. (TIF) [file pgen.1010565.s002.tif]

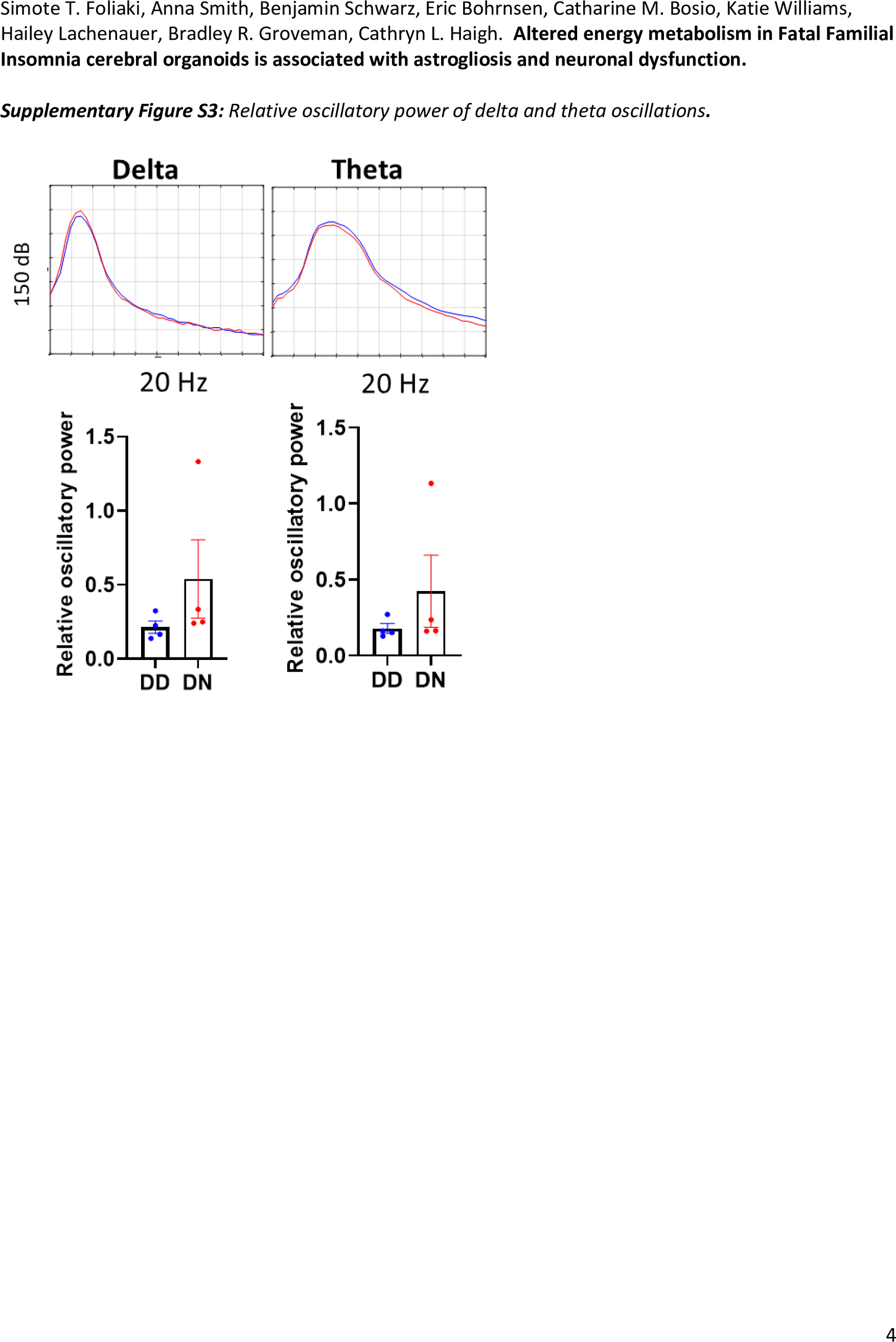

Supplement: S3 Fig — (TIF) [file pgen.1010565.s003.tif]

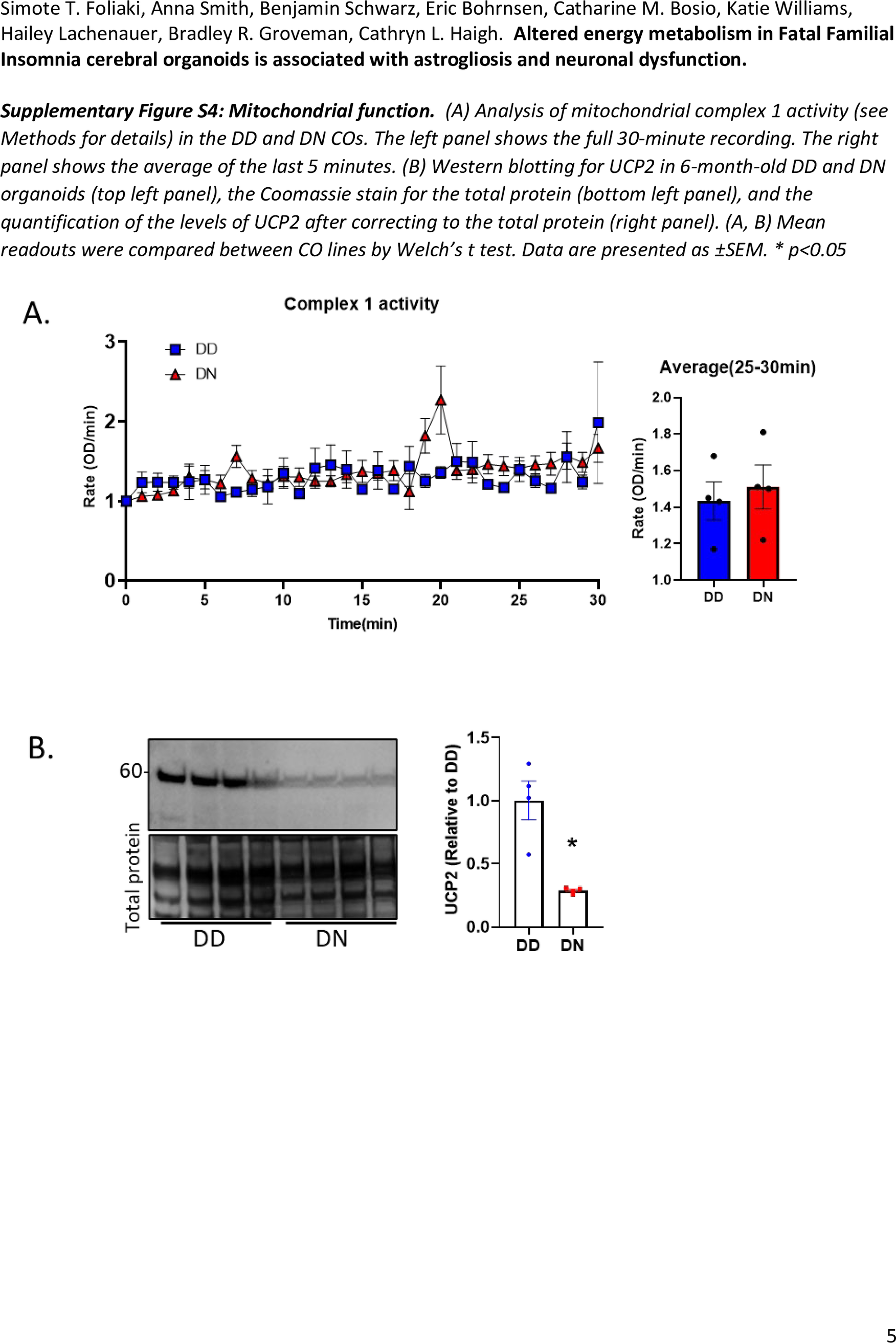

Supplement: S4 Fig — (A) Analysis of mitochondrial complex 1 activity (see Methods for details) in the DD and DN COs. The left panel shows the full 30-minute recording. The right panel shows the average of the last 5 minutes. (B) Western blotting for UCP2 in DD and DN organoids (top left panel), the Coomassie stain for the total protein (bottom left panel), and the quantification of the levels of UCP2 after correcting to the total protein (right panel). (A, B) Mean readouts were compared between CO lines by Welch’s t test. Data are presented as ±SEM. * p<0.05 Data were from 5-6-month-old COs and are presented as ±SEM. * p<0.05. (TIF) [file pgen.1010565.s004.tif]

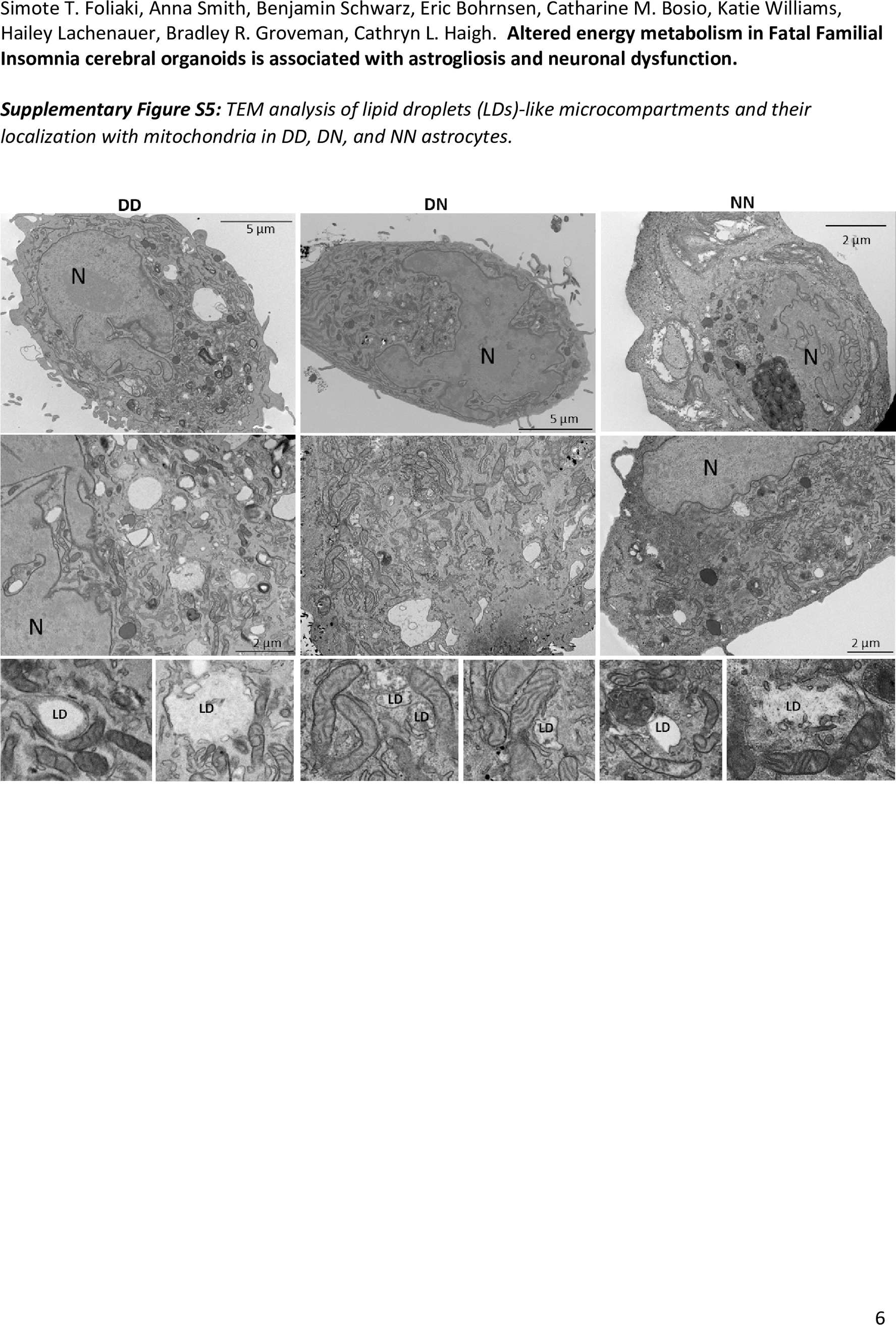

Supplement: S5 Fig — (TIF) [file pgen.1010565.s005.tif]

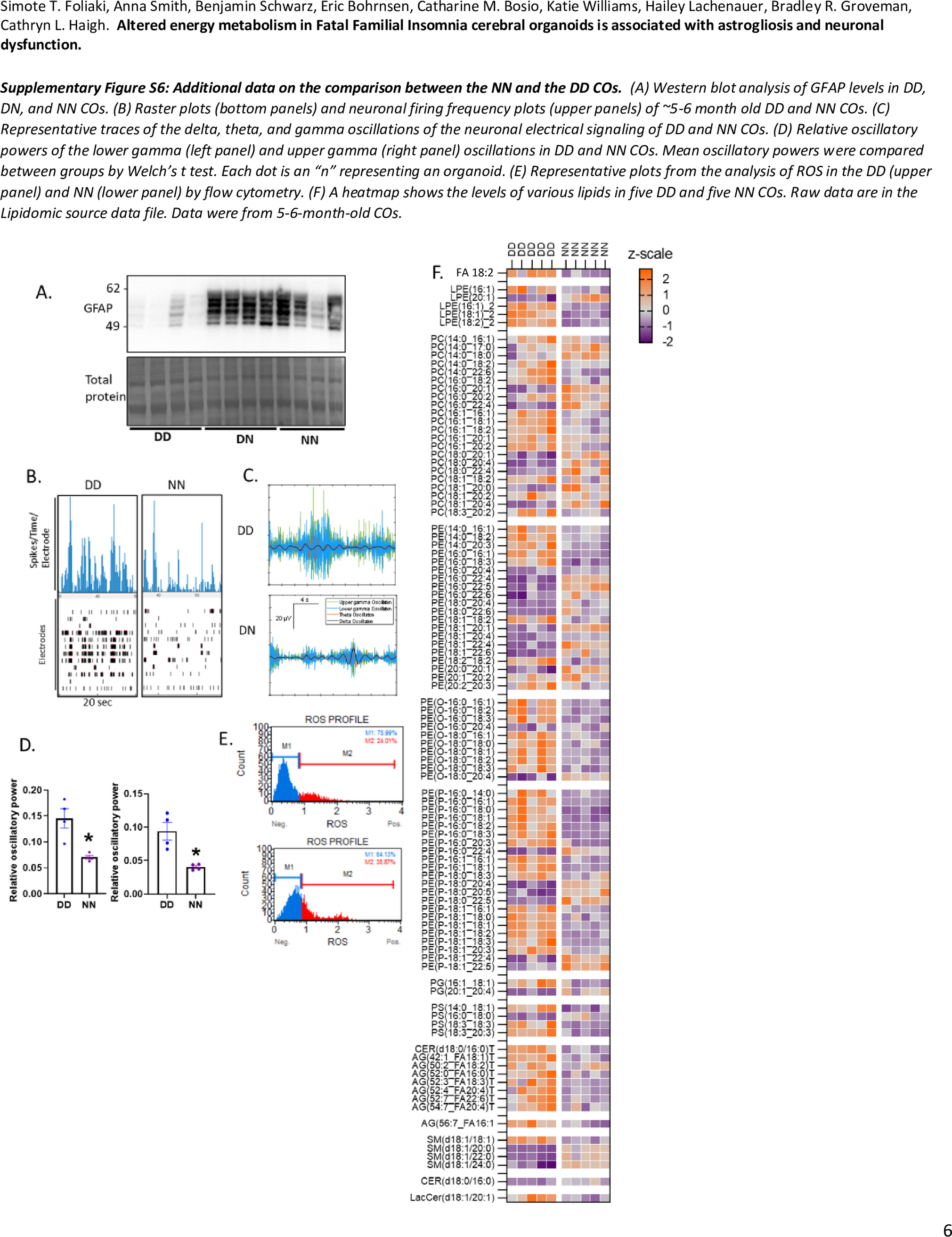

Supplement: S6 Fig — (A) Western blot analysis of GFAP levels in DD, DN, and NN COs. (B) Raster plots (bottom panels) and neuronal firing frequency plots (upper panels) of ~5–6 month old DD and NN COs. (C) Representative traces of the delta, theta, and gamma oscillations of the neuronal electrical signaling of DD and NN COs. (D) Relative oscillatory powers of the lower gamma (left panel) and upper gamma (right panel) oscillations in DD and NN COs. Mean oscillatory powers were compared between groups by Welch’s t test. Each dot is an “n” representing an organoid. (E) Representative plots from the analysis of ROS in the DD (upper panel) and NN (lower panel) by flow cytometry. (F) A heatmap shows the levels of various lipids in five DD and five NN COs. Raw data are in the S2 Dataset Lipidomic source data file. Data were from 5-6-month-old COs. (TIF) [file pgen.1010565.s006.tif]

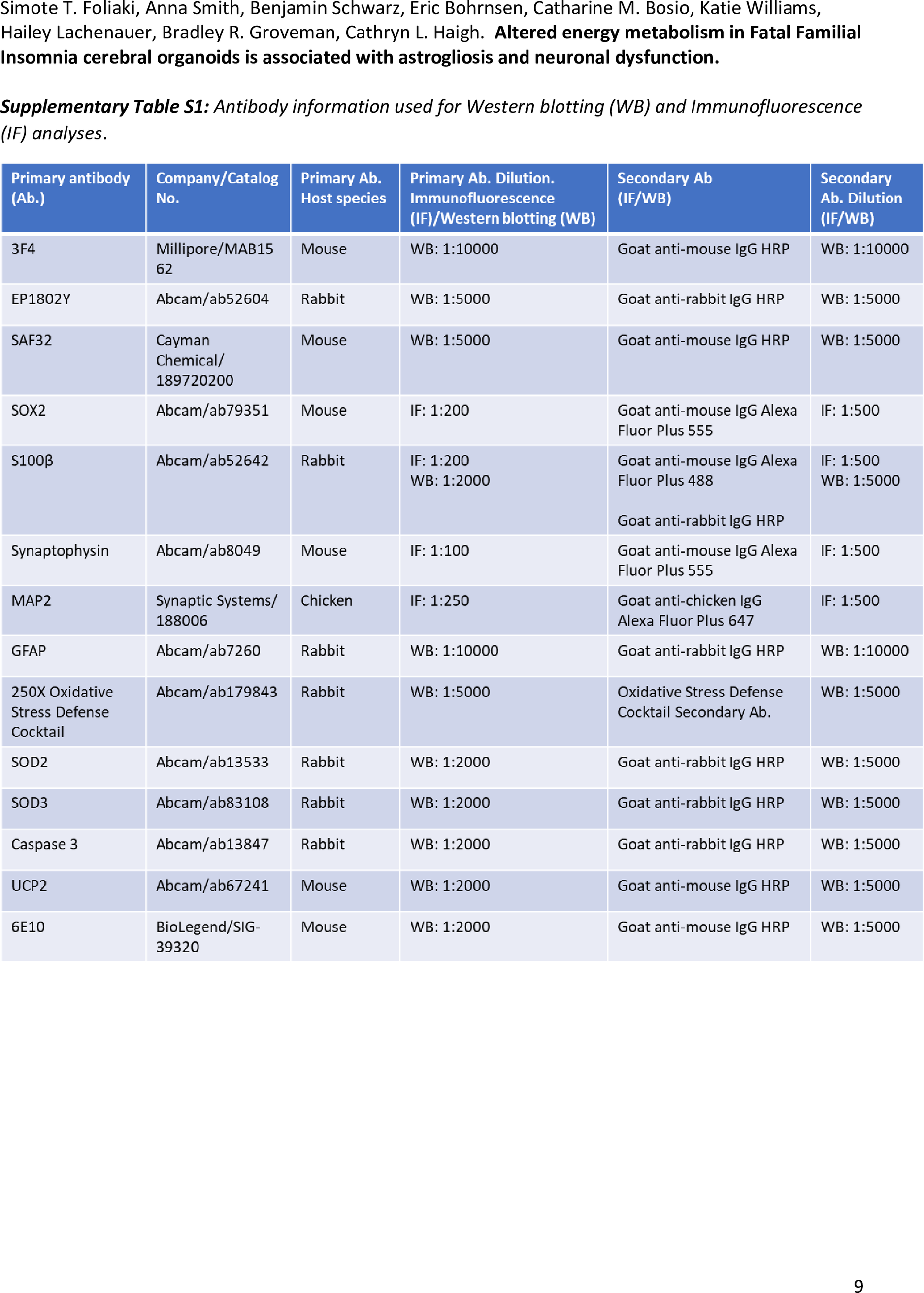

Supplement: S1 Table — (TIF) [file pgen.1010565.s007.tif]
